# Spinal cord stimulation: An emerging strategy for chronic pain relief after spinal cord injury

**DOI:** 10.4103/NRR.NRR-D-25-00553

**Published:** 2025-09-29

**Authors:** Qiwen Wang, Ying Zhang, Huifeng Zhang, Zhonghai Li

**Affiliations:** 1 Department of Orthopedics, First Affiliated Hospital of Dalian Medical University, Dalian, Liaoning Province, China; 2 Dalian Medical University, Dalian, Liaoning Province, China

**Keywords:** chronic pain, electric stimulation therapy, GABAergic neurons, nerve regeneration, neuroinflammatory diseases, neuronal plasticity, neuropathic pain, neuroprotection, pain management, spinal cord injuries, spinal cord stimulation

## Abstract

Chronic pain following a spinal cord injury refers to pain that persists or recurs after the injury. This pain can manifest as burning, stinging, or sensations similar to electric shocks. Recent studies have shown that spinal cord stimulation is an effective way to treat chronic pain after spinal cord injury. The purpose of this review is to introduce the technique of spinal cord stimulation, the clinical manifestations of spinal cord injury, and the role of spinal cord stimulation in the treatment of spinal cord injury. The mechanism and clinical application of spinal cord stimulation in the treatment of pain after spinal cord injury are discussed. The mechanism of spinal cord stimulation primarily involves three aspects: neuromodulation, neurochemical regulation, and anti-inflammatory effects, along with nerve repair. In terms of neuromodulation, spinal cord stimulation is based on the gate control theory of pain. It activates large-diameter amyloid-β nerve fibers to promote the release of inhibitory neurotransmitters by gamma-aminobutyric acidergic inhibitory interneurons in the spinal cord, thereby blocking the transmission of pain signals from small-diameter C fibers. Neurochemical studies indicate that spinal cord stimulation can regulate the balance of neurotransmitters within the spinal cord, increasing the release of inhibitory neurotransmitters such as gamma-aminobutyric acid, serotonin, and acetylcholine while reducing the levels of excitatory neurotransmitters. Additionally, spinal cord stimulation exhibits significant anti-inflammatory and neuroprotective effects, downregulating pro-inflammatory factor levels, upregulating anti-inflammatory factor expression, alleviating neuroinflammatory responses, and repairing damaged neural circuits by promoting the secretion of neurotrophic factors and axonal regeneration. Spinal cord stimulation have demonstrated remarkable efficacy in the clinical treatment of pain after spinal cord injury, but there are still limitations such as small sample size and high heterogeneity in clinical studies, as well as insufficient long-term efficacy data. Future research should conduct multi-center large-sample randomized controlled trials, and establish long-term follow-up mechanisms to improve evidence-based medical evidence.

## Introduction

Spinal cord injury (SCI) refers to structural and functional damage to the spinal cord caused by traumatic events (such as traffic accidents, falls from heights, and sports injuries) or non-traumatic factors (including tumor compression, vascular lesions, infectious diseases, and degenerative conditions) (Liu et al., 2024; Wand and Bai, 2024). This damage results in interruptions or abnormalities in the nerve conduction pathways between the brain and the body. SCI can lead to the loss of motor, sensory, and autonomic functions, with the specific impact on the patient depending on the location and severity of the injury (Deng et al., 2024). Clinical studies have shown that the higher the level of the injury (e.g., cervical injury), the worse the prognosis for the patient, potentially leading to serious consequences such as quadriplegia, respiratory dysfunction, and bowel and bladder control disorders (Hagen et al., 2011; Kandhari et al., 2022). In contrast, thoracolumbar injuries typically result in paraplegia and sensory loss in the corresponding segments (Tian et al., 2023). Importantly, patients with complete SCI are significantly less likely to recover function compared with those with incomplete injuries, suggesting the necessity of early and accurate assessment.

As the population continues to grow, the total number of SCI cases is increasing each year. Notably, the most considerable rise in the incidence of SCI has been observed among older adults, largely due to the increased prevalence of osteoporosis and a higher risk of falls resulting from decreased balance in this population. Additionally, the in-hospital mortality rate for older adult patients with SCI remains high (Jain et al., 2015). Statistics indicate that the number of new SCI cases worldwide ranges from 250,000 to 500,000 each year. In China, the incidence of SCI is also rising annually and can affect individuals of all ages (Ning et al., 2011; Reinhardt et al., 2017). The multidimensional impact of SCI extends far beyond the medical context. Research shows that up to 80% of patients with SCI suffer from chronic pain, which severely affects their sleep quality and mental health (Dombovy-Johnson et al., 2020). Functionally, SCI leads to paralysis and bowel and bladder dysfunction, significantly reducing patients’ ability to care for themselves and placing considerable burdens on both society and their families. Furthermore, patients with SCI often require long-term treatment and rehabilitation, consuming vast resources and increasing the financial burden on their families, while also putting immense pressure on the social security system. Therefore, SCI is not only an individual health issue but also a major public health and socioeconomic challenge (Badhiwala et al., 2019; Karsy and Hawryluk, 2019; Widerström-Noga, 2023).

Currently, traditional treatments for neurological dysfunction after SCI include medication, surgery, and rehabilitation (Tian et al., 2023; Izzy, 2024). However, all of these methods have considerable drawbacks and are often ineffective in controlling pain (Cragg et al., 2016). The primary purpose of surgical treatment is to reduce compression of the spinal cord and prevent secondary injury, but it is challenging to repair damaged nerve tissue (Klockner et al., 2023). Medications, such as hormones, may only be effective in the early stages of injury and have limited long-term efficacy in nerve recovery (Kupfer and Formal, 2022). In recent years, an increasing number of studies have shown that neuroregulation technologies, particularly spinal cord stimulation (SCS), remarkably improve neural function after SCI (Wu et al., 2025). By implanting electrodes to emit electrical pulses with specific parameters, SCS can promote the release of brain-derived neurotrophic factor (BDNF) and nerve growth factor (NGF), enhancing the plasticity of spinal cord nerves. SCS promotes the growth and repair of nerve axons, helping patients with SCI restore motor function, alleviate pain, and improve autonomic nerve function (Fong et al., 2009; Goganau et al., 2018; Hachmann et al., 2021; Bacova et al., 2022; Smeijers et al., 2022). Although SCS has been shown to aid in the recovery of neurological function after SCI, its specific mechanisms remain unclear. Existing studies still face challenges, including insufficient standardization of stimulation parameters, a lack of long-term efficacy data, and variability in clinical outcomes, which urgently need to be addressed through large-sample multicenter studies (Biktimirov et al., 2023; Sokal et al., 2024). Therefore, the purpose of this paper is to review the research progress on the role and mechanisms of SCS in treating chronic pain after SCI, explore potential molecular and circuit mechanisms in depth, and suggest directions for future research to provide new treatment methods for clinical practice and innovative ideas for follow-up studies.

## Search Strategy

To further understand the role and mechanism of SCS in the treatment of chronic pain following SCI, the authors used “spinal cord injury,” “spinal cord stimulation,” and “chronic pain” as keywords. A search was conducted in PubMed, Embase, Cochrane, Web of Science, and ClinicalTrials.gov. The time frame for the search extended from the inception of the databases to March 2025, with a focus on high-quality literature from the past decade. The inclusion criteria for the literature are as follows: (1) Basic research and clinical trials related to SCS in the treatment of pain after SCI, with an emphasis on the specific mechanisms and efficacy of SCS in this context; (2) Types of literature included articles or reviews. The exclusion criteria are: (1) The full text could not be obtained; (2) The content was repetitive or similar to other studies. Ultimately, 240 references were included. This review summarizes the research progress on the role and mechanism of SCS in the treatment of pain after SCI, providing a reference for the selection of clinical treatment methods and new ideas for future research.

## Overview of Spinal Cord Stimulation

### History of spinal cord stimulation technology

**Figure 1:**
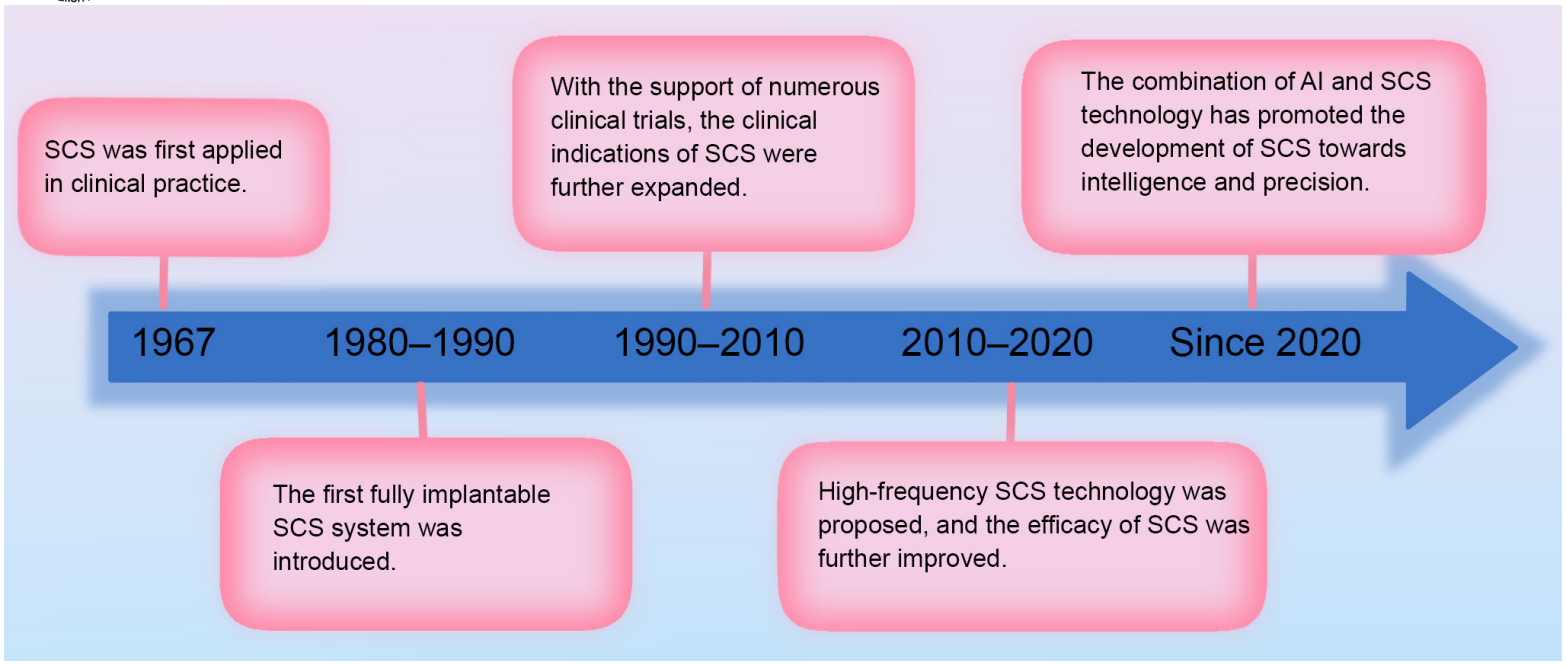
A milestone in the development of spinal cord stimulation (SCS) technology. This figure displays the timeline of SCS development: From initial clinical application (1967) to fully implantable systems (1980–1990), expansion of evidence-based indication (1990–2010), breakthroughs in high-frequency efficacy (2010–2020), and contemporary AI-driven precision neuromodulation (2020–present).

The development of SCS as a technology can be traced back to the 19^th^ to mid–20^th^ centuries (**Figure 1**). In 1965, Melzack et al. first proposed the gate control theory, suggesting that pain perception is not directly transmitted to the brain solely by peripheral injurious stimuli; rather, it is dynamically modulated by neural circuits in the dorsal horn of the spinal cord. They also explained how electrical current stimulation inhibits the transmission of pain signals. This theory revolutionized the field of pain research and directly contributed to the advancement of SCS. In 1967, Shealy et al. applied SCS for the first time in clinical practice. They performed a T2–T3 laminectomy on a 70-year-old patient with advanced lung cancer and metastatic pain in the chest and abdomen, implanting platinum electrodes into the dorsal columns of the spinal cord at the T3 level. After connecting the external stimulation device, they conducted stimulation using electrical signals with a frequency of 10–100 Hz, a current of 0.36–0.52 mA, and a pulse width of 0.5–400 ms. The effects of electrical stimulation were assessed through subjective feedback and tests of tactile and pinprick nociception in this patient. After the electrical stimulation treatment, the patient’s incisional and primary cancer pain disappeared immediately, and the effect lasted for several hours. Any subsequent pain recurrence could be rapidly controlled by adjusting the stimulation frequency, during which no anesthetic drugs were required. This trial was the first to demonstrate that SCS could relieve refractory quasi-cancer pain, marking a landmark moment that laid the foundation for the subsequent development of SCS technology.

The decade from 1980 to 1990 marked a period of rapid development for SCS technology. In 1980, the first fully implantable SCS system was introduced, dramatically increasing patient acceptance of the technology and greatly facilitating its development. Concurrently, the introduction of multi-channel electrodes and programmable pulse generators allows physicians to customize stimulation parameters for various pain areas in patients. This expansion broadens the indications for SCS beyond pure chronic pain to include peripheral nerve injury pain, complex regional pain syndrome, and other conditions (North et al., 1991, 1993). From 1990 to 2010, the clinical indications for SCS were further validated through numerous clinical trials, confirming its efficacy in conditions such as complex regional pain syndrome, diabetic peripheral neuropathy, failed back surgery syndrome, and ischemic pain. Additionally, many basic experiments revealed the specific mechanisms of SCS, providing further theoretical support for its clinical application (Cui et al., 1997; Yakhnitsa et al., 1999; Song et al., 2011). Around 2010, high-frequency SCS technology was introduced. Unlike traditional low-frequency SCS, which emits low-frequency pulses of 50–100 Hz, high-frequency SCS uses a much higher stimulation frequency of 1–10 kHz for treating chronic pain (Kasapovic et al., 2019; Isagulyan et al., 2020; Vajramani, 2020). Between 2010 and 2020, numerous studies highlighted the advantages of high-frequency SCS over low-frequency SCS in chronic pain treatment. Patients receiving conventional low-frequency SCS often experienced discomfort around the electrodes, a problem that does not occur with high-frequency SCS (Ghosh et al., 2020; Andrade et al., 2021). Some patients had poor outcomes with traditional low-frequency SCS and did not achieve effective pain control. In contrast, high-frequency SCS may be more effective for these patients, particularly those with failed back surgery syndrome and complex regional pain (Gill et al., 2019; Hasoon et al., 2023; Provenzano et al., 2023).

Since 2020, artificial intelligence (AI) technology has been gradually applied to SCS to promote the development of SCS toward increased intelligence and precision. AI can select appropriate stimulation parameters based on individual patients’ conditions to achieve personalized treatment. In recent years, AI has played an increasingly important role in the application of SCS (De Ridder et al., 2021). It can assist physicians in analyzing patients’ conditions and automatically adjusting the frequency, intensity, and other parameters of stimulation to achieve more precise treatment. AI can also integrate with imaging data to determine the optimal placement of electrodes, ensuring that they cover the key nerve pathways involved in pain transmission. By analyzing data from a large number of patients, AI can help predict the long-term outcomes of SCS, allowing patients to clarify their psychological expectations regarding treatment effectiveness (Bydon et al., 2022). Overall, AI technologies are making SCS smarter and more personalized, and they may further improve SCS efficacy and streamline the treatment process in the future (Adil et al., 2022; Hadanny et al., 2022; Hariharan et al., 2023).

### Application of spinal cord stimulation

**Figure 2:**
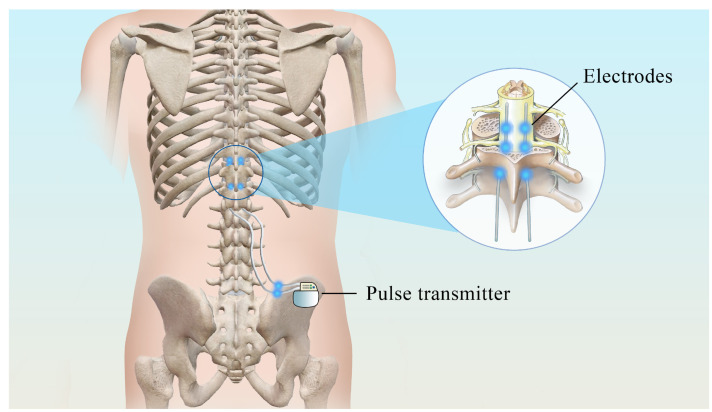
Diagram of the spinal cord stimulation (SCS) model. The SCS device consists of a pulse transmitter, wire, and electrodes that transmit an electrical signal to the spinal cord.

SCS is an advanced neuromodulation technology that modulates the transmission of neural signals by delivering electrical pulses through electrodes implanted near the spinal cord (**Figure 2**). SCS is primarily used to treat patients with chronic pain who do not respond to pharmacological interventions or conventional therapies, such as those for neuropathic pain, complex regional pain syndrome, and chronic low back and leg pain (Gill et al., 2019; Hasoon et al., 2023). Additionally, SCS shows good potential in treating SCI. By electrically stimulating spinal cord neural pathways, this technique may enhance sensory and motor functions in individuals with SCI, facilitate neural pathway remodeling, and improve patients’ quality of life. This minimally invasive surgical technique is reversible and allows the physician to adjust stimulation parameters according to the condition, thereby achieving personalized treatment for the patient and reducing the side effects of SCS (Sun et al., 2021). Data shows that the number of SCS clinical trials in the United States increased from 12,680 in 2009 to 36,280 in 2018. This demonstrates the growing utilization of SCS in the United States, with the therapy increasingly being accepted by patients as an emerging treatment option (Manchikanti et al., 2021). At the same time, more and more studies have shown that SCS has a definite effect on chronic pain after SCI in recent years (Geurts et al., 2017; Linderoth and Foreman, 2017; Chakravarthy et al., 2018; Huang et al., 2019).

### Classification of spinal cord stimulation

Currently, there is no unified classification standard for SCS in clinical practice and research, resulting in a lack of comparability between different studies and treatment methods. This inconsistency complicates the development of personalized treatment plans for patients. Establishing clear classification criteria for SCS is crucial for standardizing treatment regimens, optimizing efficacy, and advancing SCS technology. Based on recent literature related to SCS classification (Huang et al., 2019; Guzzi et al., 2022; Rahman et al., 2022; Lam et al., 2023), the authors propose a classification of SCS based on the location of electrode implantation, waveform, and stimulation frequency, aiming to provide new ideas and directions for formulating a unified classification standard (**Additional Table 1**).

**Additional Table 1:** Classification and characteristics of SCS

Classification basis	Form	Specificity
Electrode implantation position	Subdural SCS	The electrodes are placed between the dura mater and the arachnoid membranes for direct stimulation of the spinal cord with high precision.
	Epidural SCS	The electrodes are placed between the surface of the spinal cord and the dura mater, and the electrical signals are applied to the neural structures on the surface of the spinal cord for better safety and adjustability.
Stimulation waveform	Continuous pulse SCS	The electrical stimulator continuously sends periodic electrical pulses to the implanted electrodes and acts on the spinal cord nerve structures without interruption.
	Intermittent pulse SCS	Send intermittent electrical stimulation pulses, with adjustable stimulation parameters for more flexible application.
Stimulation frequency	LF-SCS	Stimulation frequencies are typically 50 to 120 Hz and are widely used in the treatment of chronic pain.
	HF-SCS	The stimulation frequency is usually 1000 Hz or higher, which does not produce abnormal sensations and is more effective.

HF-SCS: High frequency spinal cord stimulation; LF-SCS: low frequency spinal cord stimulation; SCS: spinal cord stimulation.

#### Classification according to electrode implantation location

According to the location of electrode implantation, SCS can be divided into subdural stimulation and epidural electrical stimulation (Canós-Verdecho et al., 2021). Subdural stimulation involves implanting electrodes into the subdural space between the dura and arachnoid membranes so that the electrodes are in direct contact with the spinal cord. Electrical stimulation signals can act directly on the neural structures and conduction pathways of the spinal cord. Compared with the epidural electrical stimulation, the electrical signal from the spinal canal stimulation can act on the nerve structure more directly and more accurately, thus affecting the nerve conduction and promoting the recovery of nerve function more effectively. In epidural SCS, electrodes are placed in the epidural space between the surface of the spinal cord and the dura mater. The electrical signals generated by the electrodes interact with the neural structures on the surface of the spinal cord to achieve the desired therapeutic effect (Mansour et al., 2022; Chalif et al., 2024). Although the effect of epidural electrical stimulation is not very direct compared with intraspinal stimulation, it has the advantages of low implantation risk, easy adjustment of electrode position, wide indications, and low risk of infection and complications, which makes it more commonly used in clinical work.

#### Classification according to stimulation waveform

According to the stimulation waveform, SCS can be divided into two main categories: continuous pulse electrical stimulation and intermittent pulse electrical stimulation. Continuous stimulation refers to an electrical stimulator that continuously delivers periodic electrical pulses to the implanted electrodes, acting on the spinal cord nerve structures without interruption. Continuous wave is the most commonly used waveform in SCI, which has been used clinically for many years and is widely accepted by patients (Chitneni et al., 2024). Unlike continuous stimulation, the signal from intermittent stimulation stimulates the spinal cord intermittently, and the signal is usually output in a periodic pulse train or variable pattern. Thus, the physician can individually adjust the intensity and frequency of stimulation according to the specific condition of the patient. Because intermittent pulse electrical stimulation is more flexible and easy to adjust, its clinical application is becoming more and more widespread. According to the study of Piedade et al. (2022), each waveform of SCS has its applicable scenario, and more appropriate treatment methods should be selected. In this way, the symptoms of chronic pain can be improved. At the same time, combined treatment with different waveforms is also available for patients with extremely complex conditions.

#### Classification according to stimulation frequency

SCS can be divided into low-frequency SCS (LF-SCS) and high-frequency SCS (HF-SCS) according to different stimulation frequencies. LF-SCS is a traditional SCS mode, usually with a stimulation frequency of 50–120 Hz. This mode has been used clinically for decades, particularly to help control chronic pain such as back and lower limb pain. HF-SCS is a new mode developed in recent years, typically operating at 1000 Hz or higher (Kasapovic et al., 2019; Isagulyan et al., 2020; Vajramani, 2020). It has been suggested that HF-SCS may be more effective for patients who have a poor response to conventional SCS. Additionally, HF-SCS offers advantages in terms of higher safety and fewer adverse reactions, particularly by avoiding the numbness commonly associated with traditional SCS (Hasoon et al., 2023; Provenzano et al., 2023). A detailed and standardized classification of SCS is crucial for optimizing its therapeutic effects, promoting personalized treatment, fostering technological innovation, and improving treatment safety. In the future, we should further unify the classification criteria for SCS, conduct high-quality clinical research on the different types of SCS, and focus on the diseases and symptoms applicable to each type. This approach will enhance clinical effectiveness and provide more accurate and high-quality treatment options for patients with spinal cord injuries.

## Clinical Manifestations and Treatment Strategies for Spinal Cord Injury

### Clinical manifestations

SCI can cause a variety of neurological dysfunctions, mainly involving the body’s motor regulation, sensory conduction, and autonomic nervous system. Clinical manifestations vary depending on the segment of the injury (**Additional Table 2**). The primary manifestation of motor function abnormalities is limb paralysis, and the severity of clinical symptoms depends on the extent of the injury, the affected segment, and the degree of disruption in specific conduction tracts. If the corticospinal tracts are involved (upper motor neuron injury), the patient will experience spastic paralysis. In contrast, lower motor neuron injuries caused by cauda equina damage result in flaccid paralysis with reduced reflexes below the damage plane. In terms of sensory deficits, damage to the dorsal columns often results in abnormalities in tactile discrimination, vibration perception, and proprioception. When the spinal cord is unilaterally damaged, sensory conduction in the trunk and limbs on the same side below the lesion level will be significantly affected. If the lesion involves the anterolateral region of the spinal cord (lateral thalamic fasciculus), it triggers impaired pain and temperature perception of the contralateral limb. The clinical manifestations of autonomic dysreflexia vary widely between individuals, the most prominent is dysfunctions of urination and defecation, other possible symptoms include sexual dysfunction, impaired cardiovascular function, and impaired respiratory function (DiMarco and Kowalski, 2013; Krassioukov and Elliott, 2017; Wirz and van Hedel, 2018; DiMarco et al., 2024).

**Additional Table 2:** Clinical manifestations of different segments in complete spinal cord injury

Damaged segment	Motor dysfunction	Sensory dysfunction	Autonomic dysreflexia
C1—C4	Quadriplegia with paralysis of the septum (C1-C3).	Loss of all sensation below the neck.	Respiratory dysfunction (C1-C3), bladder and bowel dysfunction, and impaired blood pressure regulation.
C5	Shoulder abduction and elbow flexion are preserved, and the hand and lower extremities are paralyzed.	Loss of sensation below the neck, with some retention of shoulder sensation.	Bladder and bowel dysfunction and impaired blood pressure regulation.
C6	Shoulder abduction, elbow flexion and wrist dorsiflexion were preserved, and the hand and lower extremities were paralyzed.	Loss of sensation below the neck, with some retention of sensation on the outside of the forearm.	Bladder and bowel dysfunction and impaired blood pressure regulation.
C7	Shoulder abduction, elbow flexion, wrist dorsiflexion, and elbow extension were preserved, and the hands and lower extremities were paralyzed.	Loss of sensation below the neck, with some retention of sensation in the back of the hands and fingers.	Bladder and bowel dysfunction and impaired blood pressure regulation.
C8	Shoulder, elbow, and wrist and finger flexion are preserved and the lower limbs are paralyzed.	Loss of sensation below the neck, with some retention of sensation in the palms of the hands and inner fingers.	Bladder and bowel dysfunction and impaired blood pressure regulation.
T1—T6	Upper limbs are normal, and lower limbs are paralyzed.	Loss of sensation below the plane of injury, including the trunk and lower extremities.	Bladder and bowel dysfunction and impaired blood pressure regulation.
T7—T12	Upper limbs are normal, and lower limbs are paralyzed.	Loss of sensation below the level of injury, including the abdomen, back, and lower extremities.	Bladder and bowel dysfunction and impaired blood pressure regulation.
L1—L2	Hip flexion may be preserved and lower limbs are paralyzed.	Loss of sensation below the plane of injury, including the anterior and medial thighs.	Bladder and bowel dysfunction and sexual dysfunction.
L3—L5	The lower extremities are partially paralyzed, and knee extension and ankle flexion may be preserved.	Loss of sensation below the plane of injury, including the lower leg and foot.	Bladder and bowel dysfunction and sexual dysfunction.
S1—S2	Partial paralysis of the lower extremities, with possible preservation of ankle extension and foot movement.	Loss of sensation below the plane of injury, including plantar, dorsal and perineal areas.	Bladder and bowel dysfunction and sexual dysfunction.
S3—S5	Lower extremity movements may be normal, with paralysis of the perineal muscles.	Loss of sensation in the perineum and around the anus.	Bladder and bowel dysfunction, and sexual dysfunction.
Cauda equina syndrome	Weakness or paralysis of the lower limbs, usually asymmetrical.	Loss of sensation in the lower extremities, perineum and anus, usually asymmetrical.	Bladder and bowel dysfunction and sexual dysfunction.
Spinocerebellar syndrome	Weakness or paralysis of the lower limbs, usually symmetrical.	Loss of sensation in the perineum and around the anus.	Bladder and bowel dysfunction and sexual dysfunction.

This table displays the motor, sensory, and autonomic nerve function symptoms associated with different injured segments in complete spinal cord injury.

Pavese et al. (2016) utilized data from the European multicenter spinal cord injury study, which included 1250 patients with traumatic SCI, for a regression analysis and grouping based on the lower extremity motor score (LEMS). The results showed that neurogenic bladder symptoms, such as urinary incontinence and incomplete bladder emptying, occurred in 93% of patients with a LEMS of 0, 50% of patients with a LEMS greater than 25%, and only 7% of those without motor deficits 1 year after injury. All patients with complete SCI and most patients with incomplete SCI experience gastrointestinal symptoms, such as neurogenic bowel dysfunction and anal sphincter dysfunction, leading to decreased movement and digestive function, as well as decreased defecation or fecal incontinence (Glickman and Kamm, 1996). Optimizing diet and using laxatives can help alleviate these symptoms and improve patients’ quality of life. If these measures are ineffective, alternative methods such as nasal feeding and surgical interventions may be considered (Emmanuel, 2019).

Many studies have shown that patients are also prone to sexual dysfunction after SCI (Hubscher and Johnson, 2000; Biering-Sørensen and Sønksen, 2001; Miranda et al., 2016). However, the related symptoms of men and women are not identical. Men mainly present with abnormal erection and ejaculation (Brackett et al., 2010). Ibrahim et al. (2016) suggest that male patients with abnormal sexual function after SCI may lead to infertility. This is mainly due to the patient’s own erectile dysfunction, ejaculatory dysfunction, and abnormal semen. For such patients, phosphodiesterase inhibitors can be used first, and if the effect is not good, penile vibration stimulation or prostate massage can be used. Women mainly present with changes in the ability to have an orgasm and a change in sexual climax time (Sipski et al., 1997, 2001), and there may also be urinary incontinence, pain, spasm, and other symptoms during sexual life, which seriously impact patients’ quality of life and mental health (Courtois et al., 2018). In a prospective study of 16 patients with SCI, Rutberg et al. (2008) investigated prolactin levels and the menstrual cycle, and found that most patients had hyperprolactinemia and amenorrhea, indicating that transient stress after SCI would lead to transient hyperprolactinemia and transient amenorrhea in patients, which would temporarily affect fertility.

Cervical or thoracic medullary SCI results in dyspnea and cough dysfunction due to weakness of the chest wall muscles and respiratory muscles and reduced chest wall compliance (Hachmann et al., 2017). A recent multicenter survey found significant restrictive ventilatory deficits in 30.9% patients with SCI and 35.9% reported cough dysfunction (Postma et al., 2016). This not only impairs respiratory function but is also a considerable risk factor for respiratory disease and death. A physiologic cough expels bronchoalveolar secretions and sputum to maintain intrapulmonary homeostasis, and this function is impaired in cough dysfunction, which, combined with sympathetic dysfunction-induced respiratory secretions and bronchoconstriction, obstructs secretion expulsion and gas transport, increasing susceptibility to pathogens (Schilero et al., 2005).

In patients with SCI, autonomic nerve damage also leads to bradycardia, low resting blood pressure, upright hypotension or abnormal acute hypertension, which can lead to patients experiencing health-related quality of life declines, reduced athletic performance, and even cognitive deficits (Krassioukov and Claydon, 2006; Peters et al., 2023). SCI (especially T6 and above) disrupts sympathetic downstream pathways, resulting in abnormal sympathetic regulation of heart and blood vessels, and the parasympathetic nerves are relatively active, which in turn triggers bradycardia, hypotension, and postural blood pressure fluctuations. Abnormal remodeling of spinal cord neurons after injury leads to sympathetic oversensitivity, and minor stimuli (e.g., bladder stimulation) can trigger abnormal autonomic reflexes, leading to abnormal acute hypertension. Prolonged sympathoinhibition also results in myocardial atrophy, persistent vasodilation, and decreased blood volume, which, in combination with chronic inflammation and metabolic disorders, increases cardiac arrhythmias, atherosclerosis, and cardiovascular disease (Krassioukov and Claydon, 2006).

### Treatment strategies

SCI can lead to multi-system symptoms, but it is often difficult to manifest in the early stage. Early diagnosis and treatment are essential to improve the prognosis of patients. When a patient has sensory and motor disorders in any part of the body after injury, we should be alert to whether the patient has SCI and immediately carry out relevant examination and evaluation. For those patients who are conscious, responsive, and able to cooperate with the examination, physical examination, computed tomography (CT), magnetic resonance imaging (MRI), and other imaging examinations can be used to diagnose the disease as soon as possible (Xu et al., 2022). For some patients with blurred consciousness who are unable to communicate and cooperate, it is of great significance to identify other early symptoms of SCI.

#### Acute stage treatment

SCI is the same as other acute central nervous system (CNS) injuries, and the neurological function of patients will gradually decrease within a few hours after injury. Therefore, it is essential to make a rapid diagnosis of patients in the stage of acute injury and provide neuroprotection-related treatment, which may completely change the prognosis and functional recovery of patients. At the same time, blood pressure and other vital signs should be closely monitored, and the most important purpose is to avoid systemic hypotension after injury, which will affect the blood perfusion of the spinal cord, and avoid aggravating injury (Ahuja et al., 2017). The 2013 guidelines for SCI proposed that that systemic hypotension should be avoided and that a mean arterial pressure between 85–90 mmHg should be maintained for 7 days to facilitate the recovery of patients (Resnick, 2013; Ryken et al., 2013). For patients with definite spinal cord compression or spinal instability, surgical treatment should be performed as soon as possible. Batchelor et al. (2013) conducted a meta-analysis that collated the results of 21 animal studies and showed that spinal cord decompression improved neurological symptoms by 35%, and that the early performance of spinal cord decompression was an important predictor of improved outcomes. From the perspective of surgery itself, segmental fixation surgery can not only fully decompress the spinal cord, but also repair the structural integrity and stability of the spine. This not only prevents persistent SCI caused by spinal cord compression, but also allows patients to perform early activities and functional exercises to enhance the efficacy of subsequent physical therapy and accelerate the recovery of patients (Wilson et al., 2017). The drug therapy in acute SCI is to inject a large amount of hormones such as methylprednisolone to reduce the edema and inflammation of the spinal cord, and reduce the complications in the later stage of SCI. Neuroprotective drugs such as naloxone may also be administered. There is substantial literature demonstrating the therapeutic effect of glucocorticoids for acute SCI (Hall and Braughler, 1982; Braughler and Hall, 1984), and hormone pulse therapy has been widely accepted by physicians. Eck et al. (2006) conducted a questionnaire to assess whether physicians use glucocorticoids to treat acute SCI, receiving responses from 305 surgeons; 85.9% of the doctors indicated that patients should be treated with corticosteroids within the first 8 hours.

#### Recovery period treatment

In the recovery period of SCI, symptomatic treatment is often performed according to the specific symptoms of the patient, such as pain, neurological dysfunction, and intestinal dysfunction. Medications that promote neurological recovery can be combined with rehabilitative treatments such as functional exercise. Minocycline has been shown to play a neuroprotective role in patients after SCI by reducing local inflammation and inhibiting oligodendrocyte apoptosis (Lee et al., 2003; Wells et al., 2003). Riluzole also provides neuroprotection and aids in restoring neurological function after SCI. As a sodium channel blocker, it prevents the continuous activation of neuronal voltage-gated sodium channels, thus preventing the swelling and death of nerve cells while reducing their excitability (Wilson and Fehlings, 2014). SCS is a reliable therapy for chronic SCI recovery. SCS not only treats chronic pain following SCI but also promotes the recovery of neurological functions, such as motor nerve function, sensory nerve function, and improvements in intestinal function and neurogenic bladder symptoms (Hachmann et al., 2021; Smeijers et al., 2022). Kapadia et al. (2024) conducted a randomized controlled study that recruited 34 patients with SCI who were randomly assigned to receive either SCS treatment or conventional treatment. The study utilized the spinal cord independence measurement (SCIM) to collect and evaluate data on relevant metrics such as gait and balance. It was found that the SCIM mobility scores of the intervention group improved continuously over time compared with the control group, and that patients’ speed, endurance, and balance while walking also improved after completing treatment. Herrity et al. (2018) carried out a controlled study in which a patient with chronic exercise-induced complete SCI was treated with SCS, and micturition was monitored over 4 months alongside four other participants. The study found that the bladder reflex emptying rate was significantly elevated in the subjects, confirming the potential of SCS to restore autonomic function and alleviate urinary symptoms such as neurogenic bladder.

## Role of Spinal Cord Stimulation in the Treatment of Spinal Cord Injury

As a neuromodulation technique, SCS acts on specific segments of the spinal cord through electrical stimulation, and shows unique advantages in the recovery of neurological function after SCI. Its mechanism of action involves the regulation of nerve excitability, the reconstruction of neural circuits, and the regulation of the cell regeneration microenvironment. Clinical studies have also confirmed its effect on the improvement of motor function and autonomic function.

### Mechanism of spinal cord stimulation to promote the recovery of nerve function after spinal cord injury

The imbalance of neuronal excitability after SCI is the core pathological feature leading to motor dysfunction. SCS precisely regulates the activity of nerve fibers in the dorsal columns of the spinal cord through low-intensity electrical stimulation, achieving a dynamic balance of neuronal excitability. When neuronal hyperexcitability triggers spasms, SCS stabilizes intracellular chloride ion concentration by maintaining the expression level of potassium-chloride cotransporter protein-2, thereby enhancing γ-aminobutyric acid (GABA)-mediated inhibitory postsynaptic potentials and effectively relieving tonic muscle contraction (Ganguly et al., 2021; Malloy and Côté, 2024). Malloy and Côté (2024) demonstrated that continuous SCS intervention for 5 days could reduce spinal muscle contraction by decreasing spinal nerve fiber activity and preventing the development of motor dysfunction in the thoracic segment. This 5-day SCS intervention elevated potassium-chloride cotransporter protein-2 protein expression in the dorsal horn of the spinal cord while reducing the frequency of spasticity episodes. For hypotonia caused by neuronal hyperexcitability after SCI, SCS works by reconstructing the spinal-muscle neural circuits. Zhou et al. (2024) used a dual-channel electrical stimulation system combined with epidural SCS and simultaneous stimulation of muscles to successfully simulate the feed-forward and feedback mechanisms of spinal sensorimotor circuits. This study showed that the neural signals generated by different electrical stimulation parameters would lead to characteristic neural circuit responses, increase the excitability of spinal neurons, and promote the recovery of motor and sensory nerve functions after SCI. The underlying mechanism may involve SCS promoting the reconstruction of neural circuits between spinal cord neurons and muscles, including the connection between sensory neurons and motor neurons, as well as the regeneration of dendritic structures of motor neurons, thus restoring muscle mobility and improving motor nerve function. SCS also increases the release of glutamate and GABA in the spinal cord. Glutamate, as the main excitatory neurotransmitter in the spinal cord, depolarizes the neuronal membrane potential by activating the corresponding receptors. GABA promotes the opening of chloride channels by activating GABA-A and GABA-B receptors, thereby hyperpolarizing the neuronal membrane potential. These neurotransmitters interact with each other to restore the excitation-inhibition balance of neurons, thereby promoting the recovery of sensory nerve function (Zhou et al., 2024).

Neurons below the plane of injury in patients with complete SCI appear to have completely lost the ability to communicate with neurons above the injury site; however, there are still residual neuronal connections at the site of injury (Dimitrijevic et al., 1984). Gad et al. (2013) used epidural SCS to intervene in rats with thoracic complete SCI and found that SCS could combine with sensory inputs from motor-related proprioceptors to regulate the spinal neural network, improve motor nerve function, and considerably enhance recovery from paralysis (Rejc and Angeli, 2019). SCS remarkably increased the frequency of spontaneous movements and enhanced robustness in paralyzed rats (Gad et al., 2013). Formento et al. (2018) found that SCS could help proprioceptors retain sensory information, enabling the organism to better control the corresponding motor neurons. This suggests that preserved proprioceptive loops are important for the restoration of motor neuron function and that these loops may enhance the effects of SCS on the restoration of motor function in patients. Future studies can further analyze how SCS activates specific signaling pathways, affects the structure and function of neural networks in the spinal cord, and reconstructs damaged neural pathways. The effectiveness and safety of SCS can also be further verified by combining animal experiments with clinical applications.

SCS can promote the differentiation of oligodendrocytes, improve the survival rate and differentiation ability of mesenchymal stem cells (MSCs) in the damaged area, and accelerate the regeneration of nerve cells. It can also prevent axonal degeneration by stimulating the regeneration of damaged neurons, enabling them to survive and promoting their regeneration, which ultimately helps restore nerve function after SCI. The main reason for the non-functional signaling of neurons following SCI is the limited axonal growth capacity of damaged neurons, the failure of axonal germination, and the impeded myelination of surviving and newly germinated axons due to scar tissue around the injury site. This results in a lack of nutritional support for the damaged neurons and demyelination, leading to axonal degeneration (Alizadeh et al., 2015). Damage or severance of axons disrupts signaling and communication between neurons, resulting in the loss of sensory and motor nerve function. Since most axons are encased in myelin, damage to the myelin sheath slows or prevents the transmission of signals across the neuron, resulting in abnormal nerve function (Stadelmann et al., 2019). The most obvious changes following SCI include damage to spinal cord tissues, limited axonal regeneration, and insufficient activation of neuronal cell regeneration, all of which are key factors contributing to impaired regeneration after axonal injury (Smith and Jeffery, 2006). Therefore, stimulating axonal regeneration and restoring the integrity of damaged neurons is particularly important. Carmel et al. (2010) found that SCS promoted accelerated axonal growth of motor neurons on the ipsilateral side of the spinal cord in mice with complete SCI, resulting in considerable axonal growth on the damaged side and the restoration of normal motor control of the limbs. Bacova et al. (2022) concluded that epidural SCS with pulsed waveforms has a protective effect on the preservation of spinal cord tissue and myelin sheaths, and that prolonged epidural SCS may contribute to the recovery of myelin sheaths after SCI, thereby inhibiting oligodendrocyte damage and promoting tissue and myelin sheath regeneration. The misdirection of axonal regeneration is one reason for the poor response of peripheral axons after SCI. Kerschensteiner et al. (2005) showed that most axons attempted to regenerate within 6–24 hours after SCI, but they tended to grow in the wrong direction, ultimately leading to symptoms such as dyskinesia. Wang et al. (2022) found that using continuous waveform, electrical stimulation can induce the directional growth of nerve axons, while pulsed waveform stimulation at the lesion site can promote bidirectional growth of nerve fibers and effectively improve motor function.

The results of several studies have shown that SCS can promote oligodendrocyte differentiation and MSC function, accelerating the functional recovery of nerve cells (Wu et al., 2011; Jing et al., 2015; Kuhn et al., 2019). Oligodendrocytes are a type of glial cells in the CNS, primarily responsible for forming and maintaining myelin sheaths around neurons. They can create myelin sheaths for multiple neuronal axons simultaneously, playing an important protective and supportive role. Additionally, oligodendrocytes are involved in regulating ionic homeostasis and providing nutritional support to neurons, which is crucial for the normal function of the nervous system (Kuhn et al., 2019). SCS promotes the differentiation of oligodendrocytes, aiding in the regeneration of myelin sheaths around damaged neurons, thereby accelerating the speed of information conduction in neurons. It also offers nutritional support and metabolic regulation for neurons, contributing to the recovery of neurological function after SCI. Jing et al. (2015) treated mice with SCI using SCS and found that the differentiation of oligodendrocyte precursor cells was considerably improved, and the secretion of neurotrophic factors was increased. This suggests that SCS can enhance the differentiation of oligodendrocyte precursor cells and promote the release of neurotrophic factors, thereby facilitating the recovery of neurological function after SCI.

Spinal cord MSCs (SC-MSCs) are a type of MSC present in the spinal cord with the potential to differentiate into neuronal cells, promoting nerve regeneration and repair after SCI. They can secrete NGF and other growth factors, which help protect and promote the recovery of damaged neurons (Cofano et al., 2019). The results suggest that the transplantation of SC-MSCs into the damaged spinal cord may facilitate the recovery of impaired nerve function; however, its application is limited by the poor graft survival rate and low neuronal differentiation rate (Cho et al., 2009). SCS, however, can improve the survival rate of SC-MSC transplantation, subsequently increasing the levels of neurotrophic factors and other trophic factors in the spinal cord, thereby promoting the regeneration of damaged neurons. Wu et al. (2011) demonstrated that, compared with SC-MSC transplantation or SCS treatment alone, the number and proportion of SC-MSCs surviving near the lesion site were considerably increased in the SC-MSC + SCS treatment group. Additionally, the neurological function of the SC-MSC + SCS treatment group was significantly restored. This suggests that SCS can enhance the biological activity of SC-MSCs, indicating that the combination of SC-MSC transplantation and SCS has a clear effect on the recovery of neurological function after SCI. In the future, SCS with different stimulation parameters can be utilized to investigate its effects on the survival rate and proliferative ability of injured neurons, allowing for the identification of optimal stimulation parameters. The specific mechanisms by which SCS promotes synapse formation and remodeling can also be explored to provide a more comprehensive theoretical basis for the clinical application of SCS.

### Clinical application of spinal cord stimulation to promote neurological function recovery after spinal cord injury

In clinical applications, SCS has been proven to have multiple beneficial effects on the recovery of motor and autonomic functions. Regarding motor nerve function improvement, SCS remarkably enhances patients’ lower limb capabilities, including walking, lower limb muscle contraction, and balance. McHugh et al. (2020) treated 10 patients with incomplete SCI using SCS and found that their walking speed improved greatly after treatment, with a 28.6% increase in the 10-meter walking test and a 29.8% increase in the distance covered during the 6-minute walking test. This suggests that SCS can restore motor nerve function in patients with SCI, considerably improving their walking ability. Kumru et al. (2024) demonstrated that multi-segment percutaneous SCS can enhance gait function, lower limb spinal cord excitability, and increase muscle strength in patients with SCI, proving more effective than single-segment or double-segment SCS. SCS can also greatly improve upper limb functions, such as movement, muscle contraction, and grip strength. Huang et al. (2022) reported that SCS considerably improved patients’ hand grip strength and grip release rates. Patients with residual grip strength of 0.1–1.5 N exhibited notable improvements in grip strength after 4 weeks of treatment, and their grip release rates were also greatly accelerated, indicating that SCS has a substantial effect on the recovery of motor nerve function following SCI. Moritz et al. (2024) conducted a prospective single-arm study involving 60 patients to assess the safety and efficacy of SCS for recovering arm and hand function in patients with SCI. The results showed that 72% of the patients experienced remarkable improvements in arm and hand function, with notable enhancements in fingertip pinch, hand grip strength, upper limb motor and sensory abilities, and self-reported quality of life. These studies collectively suggest that SCS is safe and effective in improving arm and hand function in patients with SCI. Recent clinical trials have further demonstrated that SCS has a remarkable restorative effect on motor nerve function in patients with SCI (Samejima et al., 2022; Gorgey et al., 2023; Wan et al., 2024). In clinical practice, SCS can also be combined with medications and rehabilitation exercises to enhance efficacy by integrating various therapeutic approaches.

Patients with SCI usually have symptoms related to autonomic dysreflexia, such as neurogenic bladder and bowel dysfunction. SCS treatment can effectively improve the autonomic function of patients and relieve the corresponding symptoms. Herrity et al. (2018) found that the bladder reflex emptying rate of SCS-treated patients with complete SCI increased considerably, suggesting that SCS has the effect of restoring autonomic function, This suggests that SCS has the potential to restore autonomic function and relieve urinary symptoms such as neurogenic bladder. The results of some clinical trials have also shown that SCS has a remarkable improvement effect on bowel dysfunction in patients with SCI. DiMarco et al. (2021) placed electrodes at the T9 and T11 levels in five patients with cervical SCI and bowel dysfunction and performed SCS treatment for 21 weeks. They found that SCS had a significant effect on increasing the voiding rate of the bowel in patients with complete SCI. After 21 weeks of treatment, it was found that SCS had a significant effect on increasing the rate and volume of defecation, indicating that SCS can improve the bowel function of patients with SCI. SCS can promote the recovery of sensory nerves and autonomic function after SCI, and it has an obvious effect on reducing chronic pain, numbness, neurogenic bladder, and intestinal dysfunction that occur as a complication of SCI.

SCS, as an emerging therapeutic tool, can promote the re-establishment of neural pathways in damaged areas, axonal regeneration of damaged neurons, oligodendrocyte differentiation and enhancement of MSC function by regulating the excitability of damaged neurons, and it can have a considerable improvement on motor, sensory, and autonomic functions after SCI. Meanwhile, the results of a large number of clinical trials have shown that SCS can effectively promote the recovery of motor function in patients with SCI, reduce symptoms such as neurogenic bladder and bowel dysfunction, and greatly improve the prognosis of patients. With the deepening research on SCS technology, more and more evidence in recent years has shown that SCS has definite efficacy in reducing chronic pain after SCI.

## Classification of Pain after Spinal Cord Injury

### Acute pain after spinal cord injury

Acute pain after SCI usually refers to pain that occurs immediately or shortly after an injury, and its mechanisms and clinical features are distinctly different from those of chronic pain. In the acute phase after injury (usually referred to as within 48 hours to 3 months), patients experience pain with unique clinical features and evolutionary patterns, and the generation of this pain involves multiple mechanisms: on one hand, tissue damage directly caused by trauma triggers a local inflammatory response, which is mediated by the activation of immune cells and the release of inflammatory factors. Studies have shown that the activation of microglia and astrocytes plays a key role in the inflammatory response following SCI. These cells release a variety of pro-inflammatory factors, such as interleukin (IL)-1β and tumor necrosis factor (TNF)-α, which exacerbate neuronal damage and pain (Fleming et al., 2006; Okada, 2016). Additionally, SCI itself leads to a phenomenon known as central sensitization. Central sensitization refers to the increased excitability of neurons in the spinal cord and brain, resulting in the amplification and persistence of pain signals. Research has indicated that after SCI, neurons in the dorsal horn of the spinal cord become hyperexcitable, which is associated with an increased release of neurochemicals such as glutamate, neuropeptides, and ATP (Gwak and Hulsebosch, 2011; Gwak et al., 2012).

Notably, approximately 8.2% of patients experience neuropathic pain in the acute phase, which often predicts an increased risk of chronic pain later in life (Beloeil et al., 2017; Defrin et al., 2022). Animal studies have shown that early intervention after injury can reduce neuroinflammation and alter the chronicity of pain by inhibiting glial cell activation (Tateda et al., 2017; Ishiguro et al., 2019). For example, the application of rapamycin during the acute period inhibits microglial activation after SCI, thereby reducing the development of neuropathic pain (Tateda et al., 2017). Similarly, ONO-2506 has demonstrated significant analgesic effects during the acute period by inhibiting astrocyte activation and suppressing secondary injury after SCI (Ishiguro et al., 2019). In addition, the use of cell cycle inhibitors has been shown to be effective in limiting the development and maintenance of neuropathic pain, as they can attenuate injury-induced nociceptive hypersensitivity by reducing glial cell changes (Wu et al., 2016). In contrast, lipoxin A4, an endogenous lipid mediator, has also shown promising anti-inflammatory and analgesic effects during the acute period by modulating microglial activation and TNF-α release (Martini et al., 2016). Therefore, the “time window” has become key to acute pain management, with timely intervention potentially reducing the development of neuropathic pain through multiple mechanisms.

Traditional stepwise analgesic protocols are ineffective for acute pain in SCI, and studies have shown that high doses of opioids significantly disrupt motor function and increase cell death around the lesion, leading to both acute and long-term pain (Hook et al., 2009, 2011; Woller et al., 2014; Aceves et al., 2019; Stampas et al., 2020). Currently, there are still many limitations in the use of SCS in the acute phase of pain. Its mechanism of action primarily targets the modulation of chronic neuropathic pain, while acute-phase pain is mainly dominated by tissue injury and inflammatory responses, which differ from the targets of SCS. In clinical applications, edema, the risk of infection during the acute phase, and the need for MRI examinations limit the early implantation of SCS. However, innovative therapeutic strategies have begun to receive attention in recent years. For example, artificial hibernation technology mitigates secondary injury through mechanisms such as alleviating the inflammatory response to protect neurological function (Liu et al., 2024). Although this approach has not yet gained widespread agreement, it offers new ideas for improving pain management in the acute phase.

Although SCS still faces many challenges in its application in the acute phase, its position in the full pain management of SCI may be redefined with the deeper understanding of pain mechanisms and technological advances. Future research needs to focus on clarifying the optimal time window for intervention, establishing assessment criteria specific to the acute phase, and developing accurate patient screening models. These technological innovations and clinical studies are gradually expanding the range of indications for SCS and providing new therapeutic options for the management of pain throughout SCI.

### Chronic pain after spinal cord injury

**Figure 3:**
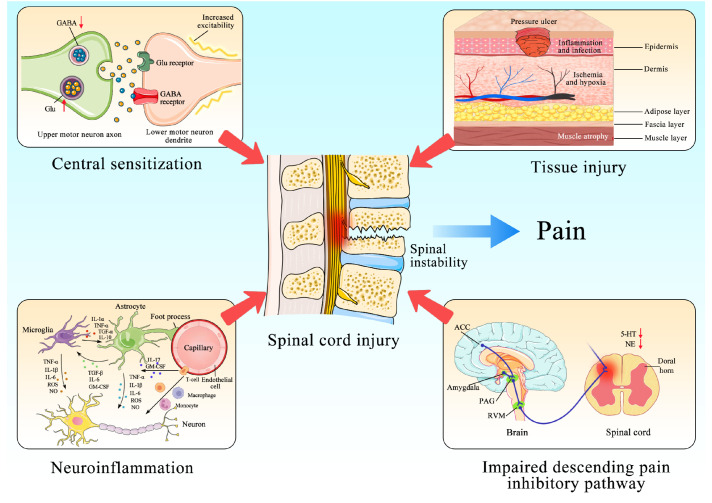
Mechanisms of chronic pain after spinal cord injury. Central sensitization, tissue damage, neuroinflammation, and abnormal pain transmission pathways contribute to the development of chronic pain. 5-HT: 5-Hydroxytryptamine; ACC: anterior cingulate cortex; GABA: gamma-aminobutyric acid; Glu: glutamic acid; GM-CSF: granulocyte-macrophage colony-stimulating factor; IL: interleukin; NE: norepinephrine; NO: nitric oxide; PAG: periaqueductal gray; ROS: reactive oxygen species; RVM: rostral ventromedial medulla; TGF: transforming growth factor; TNF: tumor necrosis factor.

Chronic pain after SCI is a common and complex complication that typically develops within months or years after the injury and persists over time (**Figure 3**). Chronic pain not only affects the physical health of patients but also considerably reduces their quality of life, leading to psychological issues such as sleep disorders, depression, and anxiety. According to statistics, approximately 70% of patients with SCI experience unilateral or bilateral chronic pain after injury (Widerström-Noga et al., 2014; Shiao and Lee-Kubli, 2018), with about one-third of these patients experiencing severe pain (Widerström-Noga et al., 2008). Chronic pain following SCI mainly includes neuropathic pain, traumatic pain, spasticity-related pain, and other pain types.

#### Neuropathic pain

Neuropathic pain is a chronic pain condition caused by damage or dysfunction of the nervous system and is the most common type of pain experienced after SCI (Finnerup et al., 2021). The underlying mechanism involves SCI causing abnormal neural pathways and central sensitization, which result in the abnormal amplification and continuous propagation of pain signals within the nervous system, leading to long-lasting and difficult-to-control pain. Previous studies have shown that glial cells, such as microglia and astrocytes in the CNS of patients with SCI, can activate immune responses, trigger neuroinflammation, and subsequently lead to neuropathic pain (Wang et al., 2022; Ying et al., 2024). To investigate the specific mechanisms underlying neuropathic pain after SCI, Wang et al. (2022) evaluated the physical condition, hind limb motor function coordination, nerve reflexes, and mechanical/thermal sensitivity pain thresholds in a rat model of SCI. After 8 weeks, parameters related to central inflammation, neurotransmission, neuromodulation, and neuroplasticity were measured. The results indicated that neuropathic pain caused by SCI was closely related to multi-point neuroinflammation, with significantly increased levels of neuroinflammatory metabolites in the spinal cord of patients experiencing neuropathic pain after SCI. Neuropathic pain in patients may occur below the level of injury, at the level of injury, or above the level of injury, each with its own characteristics (Widerström-Noga et al., 2016; Widerström-Noga, 2017). The most common type of neuropathic pain is below the level of injury, which is generally symmetrical and manifests as burning, tingling, or numbness, often in the trunk or limbs. Pain at the injury level typically presents in a ribbon-like distribution, with sensations usually described as pinprick or burning. Pain above the injury level is often associated with other neuropathic conditions, such as peripheral neuropathy, and may be caused by compression, infection, or other injuries. However, due to the complexity of pain mechanisms, some patients find it challenging to achieve relief with traditional analgesic drugs. Managing and alleviating neuropathic pain often requires a comprehensive approach, including drug therapy, physical therapy, psychological intervention, and SCS (Szok et al., 2019).

#### Traumatic pain

Traumatic pain after SCI is also a common form of pain. Unlike neuropathic pain, traumatic pain typically results from damage to non-neural tissues such as muscle, bone, skin, and joints (Mehta et al., 2013; Kataria et al., 2024). After SCI, the injured tissues release inflammatory mediators such as prostaglandins and interleukins, which can activate nociceptors in the surrounding tissue and cause pain (Luo et al., 2021; Seifert and Baerwald, 2021). Long-term inflammation and persistent damage to local tissues can lead to chronic pain. Most patients with SCI experience trauma to the spine and its supporting structures, which can contribute to traumatic pain. This pain often intensifies with ongoing spinal instability. Imaging examinations such as X-rays, CT scans, and MRIs can help determine whether the patient has spinal instability. Patients suffering from paraplegia often rely on manual wheelchairs to assist with daily activities. However, excessive or abnormal use of their limbs can lead to chronic traumatic pain in the musculoskeletal system (Dalyan et al., 1999). Chronic traumatic musculoskeletal pain can also occur when patients lose limb function. For example, in patients with quadriplegia, shoulder pain may arise from muscle atrophy around the shoulder joint and repeated dislocation of the joint (van Drongelen et al., 2006). Traumatic pain manifests in various ways, often presenting as dull pain, tingling, burning sensations, soreness, and pressure, which can considerably affect a patient’s quality of life (Cox, 2022). Ibuprofen and other anti-inflammatory drugs are commonly used to treat traumatic pain, with weak opioids such as codeine prescribed if pain is poorly controlled. Treatment may also include local physical therapy, physiotherapy, massage, and hot compresses.

#### Spasticity-related pain

Spasticity-related pain is very common after SCI, particularly in patients with cervicothoracic injuries who experience severe but incomplete motor impairment. Within 5 years of injury, approximately one-third of patients with SCI experience spasticity-related pain, and one-fifth have persistent functional limitations related to spasticity (Holtz et al., 2017). In a normal organism, upper motor neurons send inhibitory signals to regulate the activity of lower motor neurons, thereby maintaining normal muscle tone and balance. However, when SCI occurs, the inhibitory function of the upper motor neurons is compromised, leading to weakened or absent inhibitory signals. This results in the loss of control over the lower motor neurons, leaving the muscles in a state of over-excitation. The occurrence of spastic pain is also associated with increased levels of excitatory neurotransmitters, such as glutamate, and decreased levels of inhibitory neurotransmitters, such as GABA, in the spinal cord following SCI (Burns et al., 2016). At the onset of spastic pain, patients often experience intense, acute pain resembling cramping, which is usually sudden and of short duration (Bonfert et al., 2023). Spasms can cause the muscles to remain tense for extended periods, leading to limited joint movement and a persistent sensation of pain. Spasticity not only causes pain but also restricts the patient’s ability to move, affecting walking, sitting, and activities of daily living. It can even impact sleep, resulting in fatigue and emotional issues. Spasmodic pain can be treated with medications such as oral baclofen, tizanidine, tetrazepam, fomepizone, dantrolene, and clonazepam (Teasell et al., 2010). For patients with refractory severe lower limb spasticity, implantable administration of baclofen may be considered (Penn and Kroin, 1985; Saltuari et al., 1992). SCS can help relieve persistent muscle contractions, relax muscles, improve local blood circulation, and ultimately reduce the frequency and intensity of spasms. Therefore, nerve block or neuromodulation technologies such as SCS can be used in refractory cases of spasticity to help alleviate spasm-related pain that is difficult to control.

#### Other types of pain

SCI can also cause other types of pain, such as phantom limb pain or chronic pressure sore pain. Phantom limb pain refers to the phenomenon where patients feel pain in parts of a limb that no longer exist or have no sensation. It often appears weeks or months after SCI (Schone et al., 2022). Phantom limb pain typically manifests as intense stabbing, burning, or pressure sensations that may be intermittent or continuous in a part of the limb that has lost sensation. Chronic pressure ulcers, also known as chronic skin pressure injuries, are commonly seen in patients with SCI. Long-term bed rest following SCI leads to a considerable increase in pressure on local tissues, resulting in the occurrence of pressure ulcers. In patients with SCI, the most common sites for bedsores are the tip of the foot, sacrum, and heel. Patients with SCI may further increase their risk of developing pressure ulcers and aggravating existing ones due to sensory loss, weight loss, and muscle atrophy. Consequently, patients with complete SCI face more than four times the risk of developing pressure ulcers compared with those with incomplete SCI (Brienza et al., 2018). Chronic pressure ulcer pain often presents as persistent pain or deep pain that is difficult to localize. The primary mechanism involves local tissue ischemia and hypoxia, which lead to necrosis and the accumulation of metabolic waste that stimulates nerve endings. Additionally, inflammation and infection can activate pain receptors, resulting in chronic pain (Grey et al., 2006). SCS can restore motor nerve function in patients as soon as possible, enabling early movement, which is also beneficial for preventing and treating pressure ulcers. There are still some deficiencies in the current classification of post-SCI pain. For example, the existing categorization is relatively broad and does not adequately subdivide or identify certain complex types of pain. Many pain types involve both neuropathic and non-neurological factors, and the current classification methods do not fully reflect the multi-level mechanisms of pain. This limitation makes it difficult to achieve accurate treatment for different pain types. Chronic pain after SCI should be further subdivided to explore the specific mechanisms of each type and facilitate precise treatment.

## Mechanism of Spinal Cord Stimulation in Treating Pain after Spinal Cord Injury

**Figure 4:**
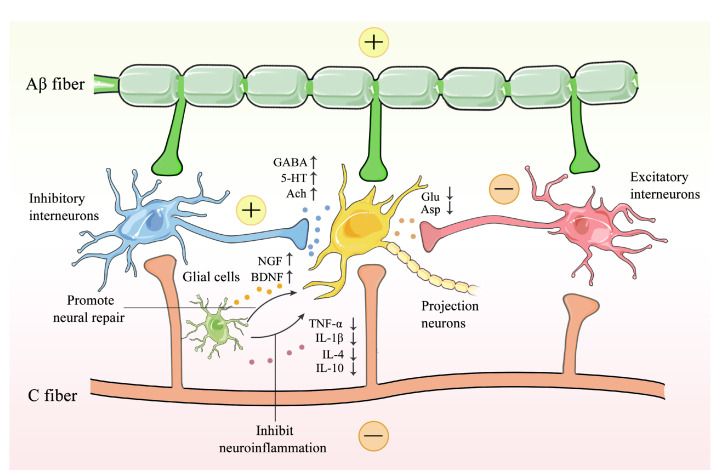
Neural activity and neurotransmitter changes in response to SCS. SCS inhibits pain signaling by modulating nerve activity, while also promoting nerve repair and reducing nerve inflammation. 5-HT: 5-Hydroxytryptamine; Ach: acetylcholine; Asp: aspartic acid; Aβ: amyloid-beta; BDNF: brain-derived neurotrophic factor; GABA: gamma-aminobutyric acid; Glu: glutamic acid; IL: interleukin; NGF: nerve growth factor; SCS: spinal cord stimulation; TNF-α: tumor necrosis factor-alpha.

As an effective method for treating chronic pain after SCI, SCS has been increasingly applied in clinical settings in recent years and has demonstrated good efficacy. However, while its role in pain relief is widely recognized, research on the specific mechanisms behind it is still incomplete. Previous studies have shown that SCS can not only affect abnormal neural activity after SCI but also regulate the pain pathway (Torsney and MacDermott, 2006; Guan et al., 2010). This therapy may alleviate pain by inhibiting pain signals, promoting the release of inhibitory neurotransmitters, and modifying the perception of pain in the brain. In this review, the potential mechanisms of SCS are briefly summarized (**Figure 4**), and the physiological mechanisms of SCS for chronic pain after SCI are discussed in greater depth in conjunction with existing research, providing more comprehensive theoretical support for clinical application.

### Neural signal regulation

#### Gate control theory of pain

Some researchers proposed the gate control theory of pain, which suggests that pain signals are not transmitted directly from the injured site to the brain (Melzack and Wall, 1965; Shealy et al., 1967). Instead, these signals are regulated through “gates” in the posterior horn of the spinal cord, which determine whether pain signals can reach the brain. Pain signals are transmitted through two types of nerve fibers: Amyloid-β (Aβ) fibers and C fibers. Aβ fibers primarily transmit non-painful tactile or pressure stimuli, while C fibers are responsible for transmitting slow pain signals. When a painful stimulus occurs, small-diameter C fibers transmit nociceptive signals to the gates, which can be opened or closed under certain conditions to determine whether the signal is further transmitted to the CNS. The opening or closing of the gate depends on various factors; for example, the activity of Aβ fibers inhibits the transmission of pain signals from C fibers, effectively “closing” the gate. This explains why rubbing or massaging the painful area can reduce pain: by stimulating Aβ fibers, the activity of C fibers is inhibited, thereby partially blocking the pain signal. This mechanism is one of the ways SCS treats chronic pain (Linderoth and Foreman, 2017).

How exactly do large-diameter Aβ fibers inhibit small-diameter C fibers? To investigate this issue, Daniele and MacDermott (2009) raised a population of transgenic mice expressing enhanced green fluorescent protein in GABAergic neurons and dissected neural structures from these mice while making continuous whole-cell recordings of the fluorescent neurons. By using electrical stimulation of the posterior horn, they observed activity at the synapses of GABAergic neurons. The results indicated that Aβ fibers could activate most of the GABAergic neurons in the dorsal horn of the spinal cord, a phenomenon that is more common in younger individuals. The study suggests that GABA-releasing inhibitory interneurons can be activated by Aβ fibers, which in turn release inhibitory transmitters to inhibit pain transmission in C fibers. This indicates that GABAergic inhibitory interneurons act as “gates” in the gate control theory. Additionally, it suggests that electrical stimulation can promote the closure of the “gate” and relieve pain. Torsney and MacDermott (2006) recorded the state of neurokinin 1 receptor (NK1R) neurons in rat spinal cord sections and used antagonists of GABA and glycine to block inhibitory neurons. The results showed that when the inhibitory neurons in the spinal cord were blocked by GABA and glycine inhibitors, NK1R neurons received more pain signals from A fibers, which in turn caused hyperesthesia. This study provided evidence that when SCS is used to treat chronic pain following SCI, it can enhance the activity of inhibitory interneurons, leading to the loss of upward conduction of pain signals in type A fibers, thereby providing analgesia. To explore the mechanism of SCS for neuropathic pain, Guan et al. (2010) ligated the L5 spinal nerve in adult male rats to simulate neuropathic pain, then used bipolar electrodes to stimulate the posterior horn of the spinal cord and observed the activity of wide dynamic range (WDR) neurons in the spinal dorsal horn. The results indicated that SCS could considerably inhibit WDR activity in neurons of the spinal dorsal horn, affecting the transmission of pain. Yakhnitsa et al. (1999) also ligated the sciatic nerve of rats to simulate neuropathic pain, and after SCS treatment, they observed the excitability of WDR neurons. The results showed that WDR activity in neurons was significantly inhibited after SCS treatment, suggesting that SCS exerts an analgesic effect by enhancing intraneuronal inhibition in the spinal cord.

Although the gate control theory appears to be quite comprehensive, it cannot fully explain the efficacy of SCS for analgesia. For example, the effect of SCS in relieving pain from acute injuries is not considerable (Kavar et al., 2000). Additionally, SCS can effectively treat pain transmitted by large-diameter Aβ fibers, which is important for pain management in patients with hyperesthesia. SCS may generate a frequency-dependent stimulus signal at the branch points of large-diameter nerve fibers, inducing a conduction block of pain information along these fibers. This suggests that SCS may not only suppress pain through simple gating mechanisms but also involve more complex processes (Cui et al., 1997; Schechtmann et al., 2008). A major challenge in studying the specific mechanisms of chronic pain induced by SCS treatment after SCI is establishing animal models that accurately reflect human SCI. Generating animal models of chronic pain using SCI methods has proven difficult because animals often struggle to recover from SCI without other complications. Therefore, the exploration of specific mechanisms of SCS largely relies on extrapolating data accumulated from previous SCS applications in patients with SCI.

#### Neurochemical mechanisms

SCS can also affect nerve activity by regulating neurotransmitters in the spinal cord, such as increasing the release of GABA, 5-hydroxytryptamine (5-HT), and acetylcholine (Ach). Cui et al. (1997) has reported that SCS can reduce excitatory neurotransmitters such as glutamate and aspartate while increase inhibitory neurotransmitters such as GABA, so that the content of excitatory and inhibitory neurotransmitters becomes balanced again. This modulation helps to reduce neuronal overexcitation, while SCS can also reduce central sensitization caused by SCI, which is the excessive response of the nervous system to pain signals. This mechanism reduces the persistence and intensity of pain by stabilizing abnormal neuronal activity and inhibiting overactive neurons.

GABA plays a role in analgesia through a variety of mechanisms. As the main inhibitory neurotransmitter, GABA binds to GABA-A receptors, resulting in the inflow of chloride ions into neurons. This increases the negative potential in neurons and causes hyperpolarization of the postsynaptic membrane, which reduces neuronal excitability and pain signaling (Qian et al., 2023). Additionally, GABA can block pain signals to the brain by inhibiting the activity of pain-transmitting neurons in the spinal cord and brainstem. Therefore, GABA effectively reduces pain by decreasing neuronal excitability and inhibiting pain signal transmission (Benke, 2022). Lind et al. (2008) injected baclofen (a GABA-B receptor agonist) into patients with neuralgia who did not respond well to SCS to determine whether SCS could remarkably improve efficacy. The results showed that SCS promoted GABA release and increased GABA concentrations in the spinal dorsal horn. The efficacy of SCS for chronic pain after SCI was considerably enhanced with baclofen use, and the effect lasted for an extended period. A retrospective study by Joosten and Franken (2020) indicated that SCS has proven to be an effective therapy for patients with drug-resistant pain following SCI. SCS is valuable in treating chronic pain by stimulating dorsal nerve roots, regulating sensory nerve excitability, and affecting the transmission of pain signals in sensory nerves. A previous study has found that SCS not only increases extracellular GABA content during testing but also that the duration of GABA elevation can be observed to extend beyond the duration of SCS. This may be related to the dysfunction of GABA reabsorption in nerve cells. Under normal conditions, nerve cells recycle excess GABA; however, this recycling ability may be weakened after nerve cell damage. Janssen et al. (2011) reported that GABAergic neuron counts decrease in the early stages of neuropathic pain but increase in the later stages in patients. This suggests that pain may be related to GABA release and the impairment of GABA receptor function. Therefore, the mechanisms by which SCS alleviates chronic pain through the GABA pathway are not yet fully understood and require further exploration in the future.

5-HT is an important neurotransmitter that helps regulate mood, sleep, appetite, and pain perception. In the process of pain signal transmission, 5-HT acts on receptors in the spinal cord and brain, blocking pain signaling and playing an analgesic role. To investigate changes in the 5-HT suppression system in neuropathic pain caused by SCI, Liu et al. (2010) performed spinal nerve ligation in adult male rats and assessed the rats’ hyperalgesia while measuring the levels of 5-HT and its metabolite 5-hydroxyindoleacetic acid in spinal cord tissue. The results indicated that in rats with spinal nerve injury, the inhibitory effect of 5-HT and its receptor agonist on the C fibers of WDR neurons was significantly weakened, and the levels of 5-HT and 5-hydroxyindoleacetic acid in the nerve tissues were also considerably reduced. These findings suggest that SCI may lead to abnormalities in the 5-HT system, which may contribute to the occurrence of chronic pain. Song et al. (2011) pointed out that the mechanism of SCS treatment in relieving neuropathic pain is closely related to the 5-HT2A, 5-HT3, and 5-HT4 receptors. Among these, the activation of the 5-HT3 receptor may exert an inhibitory effect through GABAergic interneurons, thereby providing an analgesic effect. Additionally, this study suggests that using drugs targeting these specific 5-HT receptors may enhance the analgesic effect in patients with chronic pain, offering a new strategy for the clinical application of SCS.

Acetylcholine (ACh) can produce analgesic effects by activating both M-type (muscarinic) and N-type (nicotinic) cholinergic receptors. Additionally, acetylcholine can regulate neuronal excitability in the spinal dorsal horn to inhibit the abnormal amplification of pain signals, ultimately achieving multi-level analgesic effects (Sullere et al., 2023). Zhang et al. (2009) reported that SCS promotes acetylcholine release, which then acts on acetylcholine receptors on GABAergic interneurons. The activated interneurons release GABA to prevent the transmission of pain signals. Thus, SCS can alleviate pain in patients by enhancing the secretion of acetylcholine.

### Regulation of inflammation and neural repair

After SCI, inflammation may occur in local nerve tissue, exacerbating pain. SCS can alleviate pain caused by inflammation by regulating the local immune response and inflammatory response while reducing neuroinflammation. de Geus et al. (2023) used SCS to continuously stimulate the spinal cord for 48 hours and analyzed the inflammatory balance of the spinal cord based on RNA expression of inflammatory factors such as TNF-α, IL-1β, IL-4, and IL-10. The results showed that the inflammatory response after SCI was considerably reduced following SCS treatment, confirming that SCS could alleviate pain caused by inflammation by reducing neuroinflammation. To explore the action mechanism of HF-SCS in pain relief, Yu et al. (2024) genetically manipulated the SCS-mediated signaling axis in pain through intrathecal injection of a small viral system. They demonstrated that HF-SCS reduced immune responses in the spinal posterior horn by inactivating the Kaiso-P2X7R pathological axis in microglia, allowing patients to achieve lasting pain relief.

SCS, which delivers electrical impulses to the spinal cord through implanted electrodes, can promote the release of neurotrophic factors, such as BDNF and NGF, that are essential for axon growth and repair. Additionally, SCS can enhance the neural plasticity of the spinal cord, enabling damaged nerves to recover function by reorganizing and forming new axons. This axonal regeneration helps restore normal nerve signaling and reconstruct damaged neural circuits, thereby relieving chronic pain in patients. Bacova et al. (2022) performed epidural SCS treatment in mice with SCI to determine whether SCS could stimulate and promote the growth of sensory and motor nerve fibers. The results showed that the protein levels of neurofilament light chain, growth-associated protein-43 (a marker of newly sprouting axons), and granin basic protein were considerably increased after SCS treatment, indicating that SCS plays a remarkable role in promoting the regeneration of sensory nerves in mice. Goganau et al. (2018) found that SCS treatment could accelerate the regeneration of sensory neuron axons in the spinal dorsal horn and that SCS had a time-dependent effect on promoting sensory neuron axon growth without adversely affecting perception, thus achieving the goal of chronic pain relief.

## Clinical Application of Spinal Cord Stimulation to Treat Chronic Pain after Spinal Cord Injury

In recent years, SCS has emerged as a promising neuromodulatory method for the treatment of refractory chronic pain following SCI. Although its efficacy depends on various factors, such as pain characteristics and neurological status, advances in stimulation modalities and implantation techniques have expanded its clinical applications. We synthesized the current evidence on the application of SCS for post-SCI pain, analyzed the limitations of its use and their possible causes, discussed potential future directions, and critically assessed the complications and safety issues associated with SCS in this population (**Additional Table 3**).

**Additional Table 3:** Clinical studies of SCS for the treatment of pain after SCI

Team	Type of study	Sample size	Type of pain	Treatment	Follow-up time	Main results	Adverse event
Biktimirov et al., 2023	RCT	33	Spasticity- related pain	SCS	12 mon	After 12 mon of follow-up, patients reported considerably less pain after SCS, as indicated by their VAS scores. Additionally, notable improvements were observed in reducing muscle tone, alleviating spasticity, relieving anxiety and depression, and improving motor function.	The SCS group reported a case of a pressure sore near the electrode placement site complicated by sepsis, which led to removal of the device.
Choi et al., 2019	Pilot study	10	Neuropathic pain	Single percutaneous direct current SCS	2 h	NRS, PGA, and PPI scores all decreased.	Most participants felt tingling at the electrode placement site, but it was only transient and tolerable.
Lee et al., 2009	Case report	1	Neuropathic pain	SCS	8 mon	ODI reduced from 58% to 30%, MPQ score from 40 to 20, VAS score from 9 to 1, and BDI score from 7 to 0.	None.
Kim et al., 2010	Case report	1	Neuropathic pain	SCS	9 mon	On the MPQ, the PRI was from 57/75 to 21/75, PPI from 5/5 to 3/5, functional disability was from 62/70 to 43/70.	Not explicitly mentioned.
Shu et al., 2016	Case report	1	Neuropathic pain	SCS and microsurgical DREZotomy	12 mon	The tingling pain in the left lower extremity disappeared immediately after surgery, and the pain in the left heel and right lower extremity was also successfully relieved after the activation of the SCS. One year after implantation, the patient's pain relief remained effective, and her neurological function was stable.	Not explicitly mentioned.
Reck et al., 2017	Case report	1	Neuropathic pain	Burst SCS	3 mon	The NRS score decreased from 7/10 to 4/10, reflecting a reduction in both the frequency and intensity of pain episodes, and this effect persisted at the 3-mon follow-up.	Not explicitly mentioned.
Reddy et al., 2019	Case report	1	Neuropathic pain	SCS	9 mon	The NRS score decreased from 8/10 to 2/10, indicating a 70% improvement in neuropathic pain. The patient also reported enhancements in mood, sleep quality, and overall quality of life.	Not explicitly mentioned.
Yoon et al., 2021	Case report	2	Neuropathic pain	Burst SCS	1-3 yr	One patient experienced considerable postoperative pain relief with no reduction in efficacy after 1 year of follow-up. The other patient achieved two-thirds pain relief within 6 mon after SCS, but this effect diminished over time. Reapplication of SCS 3 years postoperatively resulted in a one-third improvement in pain.	Not explicitly mentioned.
Lee et al., 2021	Case report	2	Neuropathic pain	Burst SCS	6-9 mon	The NRS score decreased from 7 to 3-5 in one patient and from 9-10 to 2-3 in another patient.	None.
Rosales et al., 2022	Case report	1	Neuropathic pain, spasm- related pain	SCS	6 mon	Symptoms improved by more than 80%, with the NRS score dropping from 10 to 1 at 1 wk after implantation. After 6 mon of follow-up, the NRS remained at 4.	None.
Fukaya et al., 2022	Case report	3	Neuropathic Pain	SCS or differential target multiplexed stimulation	1-3 yr	Three patients reported reductions in pain on the VAS, with scores decreasing from 8 to 4, from 9 to 2, and from 7 to 2, respectively. Additionally, the patients noted a decrease in their use of pain medication, relief from numbness in both lower extremities, and improvements in gait disturbances. These effects lasted for 1 to 3 yr.	Not explicitly mentioned.
Jablońska et al., 2024	Case report	8	Neuropathic pain	Burst and HF-SCS	12 mon	Two patients obtained pain relief to their satisfaction.	None.
Lee et al., 2024	Case report	1	Neuropathic pain	SCS	18 mon	Patients felt 50% to 70% pain relief when the stimulator was activated.	Not explicitly mentioned.
Park et al., 2025	Case report	1	Neuropathic pain, phantom pain	HF-SCS	6 mon	At 4 d after SCS activation, phantom limb pain was completely eliminated, and back and neuropathic pain were reduced by 30%. Quality of life also improved, and this enhancement persisted at both the 6-wk and 6-mon postoperative follow-ups.	Not explicitly mentioned.
Suarez et al., 2025	Case report	1	Neuropathic pain	SCS	2 wk	The patient reported complete pain relief and stopped taking all pain medication. Additionally, spasticity was improved by 60%, and upper extremity mobility was increased by 50%.	Not explicitly mentioned.

This table presents information and main results of clinical trials on the treatment of pain after SCI using SCS. DREZ: Dorsal root entry zone; HF-SCS: high frequency spinal cord stimulation; MPQ: McGill Pain Questionnaire; NRS: numeric rating scale; ODI: Oswestry Disability Index; PGA: patient global assessment; PPI: present pain intensity; PRI: pain rating index; SCI: spinal cord injury; SCS: spinal cord stimulation; VAS: visual analog scale.

### Current status of clinical research

To the best of our knowledge, there is a lack of large-scale RCTs specifically investigating the application of SCS for post-SCI pain. This pain condition has only been mentioned in a small-sample study (*n* = 33) focusing on improving the quality of life for patients with spasticity after SCI (Biktimirov et al., 2023). Biktimirov et al. (2023) randomized 33 patients with SCI into two groups: one receiving SCS and the other receiving intrathecal baclofen treatment, with a follow-up period of 12 months after treatment. According to Visual Analog Scale scores, patients in the SCS group showed a considerable reduction in pain, along with decreased muscle tone, alleviation of spasticity, relief from anxiety and depression, and improvements in motor function. However, the SCS group did not demonstrate a considerable advantage over the baclofen group. It is noteworthy that the baclofen group required approximately five hospital visits for baclofen pump refills during the 12-month postoperative follow-up period, while no adjustments were needed after the implantation of the SCS system. Furthermore, the incidence of adverse events was substantially lower in the SCS group compared with the baclofen group. Choi et al. (2019) conducted a single-blinded, randomized, crossover pilot study in which they administered a single percutaneous direct current stimulation to 10 patients with chronic neuropathic pain following SCI. Choi et al. (2019) measured pain indicators at the end of stimulation, as well as 1 and 2 hours afterward, and found that the Numeric Rating Scale (NRS), patient global assessment, and present pain intensity all decreased; however, these results were not statistically significant. Choi et al. (2019) suggested that the frequency, duration, and number of SCS sessions, along with the duration of the patient’s SCI, may affect treatment efficacy. They also recommended refining the phenotypic classification of neuropathic pain to better assess the effects of percutaneous spinal cord direct current stimulation.

SCS has demonstrated a good analgesic effect in some case reports. Lee et al. (2009) reported on a patient who experienced no pain relief after receiving multiple medications and other treatments. This patient chose to receive SCS and was followed up for 8 months, during which a considerable analgesic effect was observed. Kim et al. (2010) performed SCS on C1–C3 for a post-SCI pain patient due to myelitis. This participant reported a decrease in all pain scores and expressed satisfaction with the analgesic effect 9 months after receiving SCS. Shu et al. (2016) combined SCS with microsurgical dorsal root entry zone otomy in a patient with incomplete SCI, resulting in pain relief after the procedure. The analgesic effect remained good, and nerve function was stable at the 1-year postoperative follow-up. In the case report by Reck et al. (2017), a patient with complete SCI also experienced considerable pain relief with Burst SCS, with reductions in both the frequency and intensity of pain. These positive effects persisted for 3 months of follow-up. Reddy et al. (2019) reported cases that got 70% pain relief after SCS and reported the ability to sleep through the night, return to light exercise, and improved mood after treatment, with an overall satisfaction of > 80%. Yoon and Kim (2021) reported two patients with neuropathic pain after SCI, who had improvement in their pain after application of burst SCS, one patient had no reduction in efficacy after 1 year of follow-up, but the other had a reduction 6 months after surgery. In a case report of Lee et al. (2021), both patients achieved pain relief without adverse events with burst SCS, and the patient’s psychological symptoms such as depression improved. Rosales et al. (2022) reported a patient with baseline NRS score of 10/10, which was reduced to the level of 1/10 at 1 week after treatment with SCS and got significant pain improvement, and after 6 months of follow up NRS was 4/10 with 50% pain relief, which remained at the effective level, with no adverse events occurring during this period. Fukaya et al. (2022) reported three patients with neuropathic pain after SCI, in which one was treated with conventional SCS, and the other two patients using differential target multiplexed stimulation, all three patients received considerable pain improvement and reported reduced use of analgesics, improved limb numbness, and improved gait disturbance after SCS, the investigators concluded that differential target multiplexed stimulation appears to have better results than conventional SCS. Lee et al. (2024) reported on patients who felt 50% to 70% pain relief upon stimulator activation that lasted for 18 months. In this report, the researchers used a retrograde (from caudal to rostral direction) surgical implantation of the electrodes to deal with implantation limitations due to the presence of extensive epidural fibrosis at the electrode implantation site. Park et al. (2025) conducted HF-SCS on a patient experiencing phantom limb pain, neuropathic pain, and back pain following SCI. As a result, the phantom limb pain disappeared, and both neuropathic pain and back pain considerably improved, with these effects persisting at the 6-month follow-up. Suarez et al. (2025) reported a case of neuropathic pain accompanied by spasms. After SCS treatment, the pain was completely relieved, and there was considerable improvement in both spasticity and motor function.

However, there are also case reports indicating that SCS did not yield satisfactory outcomes for all patients. In previous studies (Jabłońska et al., 2024; Sokal et al., 2024), burst and HF-SCS were administered to eight patients with neuropathic pain. Unfortunately, only two of the eight patients experienced satisfactory pain relief, while the remaining six did not show notable improvement in their pain.

### Efficacy issues

SCS has been used in recent years to treat a variety of refractory chronic pain and has shown good results; however, its application in SCI is still limited by the complexity of the pathophysiological mechanisms and the lack of clinical evidence. In several case reports mentioned above, SCS reduced patients’ pain levels, but the results do not always appear satisfactory (Jabłońska et al., 2024; Sokal et al., 2024), nor did it demonstrate an advantage in the few prospective trials conducted (Choi et al., 2019; Biktimirov et al., 2023). These findings were derived from small-sample, single-center studies with varying standards of efficacy assessment, which seriously undermine the generalizability of the results, highlighting the urgency of establishing a standardized, phenotype-specific efficacy assessment system. SCS, in combination with other therapeutic regimens, has shown considerable potential in improving the efficacy of chronic pain management after SCI. For example, SCS, when used alongside conventional medical management, has been shown to notably reduce pain intensity and improve patients’ quality of life and functional capacity (Zhou et al., 2023).

In terms of technique, individualizing stimulation parameters is a core strategy to enhance the efficacy of SCS. This necessity arises from the heterogeneity of pain mechanisms and the uniqueness of neuroanatomy following SCI. One study analyzed 127 patients with central pain after SCI and found that pain types could be categorized as follows: persistent pain (95%), intermittent pain (31%), and evoked pain (45%), which includes allodynia, hyperpathia, and hyperesthesia. Different pain types have distinct mechanisms and respond differently to SCS. Spinal cord or brain stimulation has been noted to lead to a reduction in persistent pain in 36% of patients, with 0% reduction in intermittent pain and 16% reduction in evoked pain (Tasker et al., 1992). This suggests that clarifying pain phenotypes and associated mechanisms, and targeting SCS treatment accordingly, is a viable approach to improving efficacy. Spatially selective stimulation or directional selective stimulation modes can optimize the spatial distribution of electrode contacts among anatomical targets, contributing to precise anatomical localization (Canna et al., 2021; Cuellar et al., 2024). Monitoring spinal cord activity during SCS with functional MRI (Laakso et al., 2021) allows for the dynamic optimization of spatial electrode locations and current vector direction, ensuring that the stimulation field precisely covers key pain transduction nodes. In response to the dynamic changes in neuroplasticity and tissue interfaces, a closed-loop adaptive system can monitor the charge changes generated by variations in the thickness of the dura mater and the dorsal layer of cerebrospinal fluid, which can result from changes in the patient’s posture and physiological movement. This system provides real-time feedback and automatically adjusts stimulus patterns to achieve individualized stimulation parameters (Lam et al., 2023; Muller et al., 2024). Prospective clinical studies have shown the good analgesic effects of the closed-loop SCS system (Mekhail et al., 2020; Brooker et al., 2021).

In the future, AI may be combined with machine learning algorithms to set adjustable electrode positions and stimulation currents (Spieker et al., 2024), breaking through traditional models and providing data-driven decision support for individualized parameter configurations. Ultimately, this could advance SCS toward precise regulation at the level of neural circuits.

Diminishing efficacy over time is a problem that cannot be ignored (Rosales et al., 2022; Yoon and Kim, 2021) and is particularly common in the field of SCS for chronic pain management (Dones and Levi, 2018; Viswanath et al., 2019). Electrode fibrosis is a frequent issue in patients treated with SCS due to foreign body reactions and prolonged stimulation, which increase the resistance between the electrodes and the spinal cord, thereby reducing current transfer efficiency. Studies have shown that epidural fibrosis is strongly associated with pain persistence and severity and may diminish the efficacy of SCS by affecting its electrical stimulation conduction path (Cicuendez et al., 2012; Guner et al., 2021). Currently, modifications to electrode materials aimed at reducing foreign body reactions, low-intensity physiological stimulation shortly after implantation to prevent fibrosis from spreading, and the design of neural interfaces and mechanical structures that match the modulus of elasticity of the surrounding tissues or adhere to the theory of electrode miniaturization are promising research directions to address the fibrosis problem (Taccola et al., 2020). Although these methods are still in the developmental stage, there is potential for future innovations in materials and technologies to improve the durability and flexibility of electrodes and reduce fibrosis.

Maladaptive neural remodeling and central sensitization are major factors contributing to the diminished efficacy of SCS. Studies have shown that neuronal hyperexcitability and central sensitization following SCI result in a persistent increase in pain—a phenomenon that plays a key role in the development and maintenance of pain after SCI (Park et al., 2018; Gwak and Hulsebosch, 2011). Prolonged SCS may induce compensatory adaptations in the spinal cord and higher centers, leading to changes in synaptic plasticity and reorganization of neural circuits, which can result in increased pain and decreased efficacy of SCS (Latremoliere and Woolf, 2009; Ma et al., 2024). Factors related to the SCS system may include the inability of conventional open-loop SCS systems to adjust parameters in real time according to neural activity, creating a disconnection between stimulation and physiological demands, and electrode displacement, which can lead to reduced stimulation accuracy. To address the issue of diminished efficacy, it is essential to clarify the pain phenotype and underlying mechanisms for targeted treatment, as well as to implement closed-loop adaptive systems. Additionally, a previous study has indicated that combining SCS with high-frequency stimulation can provide notable pain relief, especially when the effects of conventional SCS are diminished (Andrade et al., 2021). Multi-waveform combination therapy is considered an effective option that should be explored before resorting to the removal of the SCS system. Furthermore, it has been found that the width of the preserved ventral tissue bridge correlates with the appearance and progression of neuropathic pain, suggesting that this bridge can serve as an imaging biomarker for monitoring pain progression. It may also be used to stratify patients in intervention trials and identify a superior responding group to avoid attenuation of therapy efficacy (Pfyffer et al., 2020). In conclusion, to prevent attenuation of efficacy over time, a shift from unidirectional stimulation to adaptive modulation, along with a combination of multimodal intervention strategies, is necessary to achieve precise and dynamic modulation of chronic pain following SCI.

### Complications and safety

SCS, as a reversible neuromodulation technique, offers considerable clinical benefits for many patients with chronic pain due to its minimally invasive and modifiable nature. However, complications associated with long-term implantation need to be evaluated with caution (**Additional Table 4**). The incidence of complications after SCS varies considerably across studies, with a complication rate of 31.9% reported by Kumar et al. (2006) and an incidence of up to 43% reported by Taylor et al. (2005). Despite these high rates in the current literature, it is worth noting that life-threatening complications are rare (Slangen et al., 2014). Electrode migration is the most common complication of SCS treatment and can alter stimulus conduction, resulting in reduced efficacy; in severe cases, it may necessitate surgical re-implantation of the electrode. A case review by Mekhail et al. (2011) reported an electrode migration rate of 22.6%, along with common complications such as electrode attachment failure (9.5%), electrode fracture (6%), and infection (4.5%). Cameron et al. (2004) also noted a high rate of electrode displacement (13.2%). One study found that using the dorsal median crease for “midline anchoring” of the electrodes led to a notable reduction in lead displacement rate (from 24% to 7%) (Tomycz et al., 2012). Another study employing this method achieved significant results (Mironer et al., 2008). Bendersky and Yampolsky (2014) reported infection rates ranging from 2.5% to 14% and electrode breakage rates ranging from 3% to 9%. Additionally, there are some rare complications, such as epidural hematoma (0.19%–0.32%), cerebrospinal fluid leakage (0.3%–7%), discomfort or pain around the implanted pulsatile transmitter (0.9%–12%), and intra-operative nerve injuries, which, although infrequent, require attention and proactive management (Cameron, 2004; Bendersky and Yampolsky, 2014; West et al., 2023).

**Additional Table 4:** Recommendations for the prevention and management of complications associated with spinal cord stimulation

Complication	Reference incidence	Prevention and treatment recommendation	Reference
Electrode shift	11.3%—22.6%	The electrodes were anchored to the deep fascia or dura mater using the dorsal median crease for midline anchoring. Patients were advised to avoid strenuous exercise, and surgical reimplantation was required in cases of significant displacement that affected the efficacy of the treatment.	Cameron, 2004; Turner et al., 2004; Kumar et al., 2006; Mekhail et al., 2011; Speltz Paiz et al., 2023
Electrode connection failure	0.5%—9.5%	Ensure that the device matches and that the connection is intact, the wire length is appropriate, and avoid bending. If the connection fails after programmed adjustments, surgical intervention may be necessary.	Cameron, 2004; Mekhail et al., 2011
Electrode breakage	3%—9%	Select electrodes that are highly flexible, anti-fatigue, and suitable for the patient's anatomical structure. Place them precisely and secure them firmly during the operation to avoid mechanical stress. Advise the patient to avoid strenuous exercise, and replace the electrodes in the event of breakage.	Akmal and Eljamel, 2008; Mekhail et al., 2011; Bendersky and Yampolsky, 2014
Infections	2.5%—14%	Prophylactic antimicrobial therapy, strict asepsis, percutaneous implantation of electrodes instead of surgical implantation, prophylactic postoperative antibiotics, and close monitoring of care are essential. In cases of severe infection, device removal may be necessary.	Kumar et al., 2007; Mekhail et al., 2011; Bendersky and Yampolsky, 2014; Labaran et al., 2020
Discomfort or pain at the implant site	0.9%—12%	Choose a pulse transmitter that fits the patient's size and anatomy, and avoid implanting it in bony prominences or areas prone to compression. If pain is not relieved, consider re-implantation at a different site.	Barolat et al., 1989; Law, 1995
Cerebrospinal fluid leakage	0.3%—7%	Preoperatively, high-risk patients were identified through imaging of the spinal anatomy. Minimally invasive access or image guidance was chosen for complex cases, and precise intraoperative maneuvers were performed. If cerebrospinal fluid leakage occurred, the maneuver was stopped immediately, and the need for repair was assessed. Postoperatively, patients rested in a flat position and were closely monitored.	Cameron, 2004; Garg and Wang, 2023
Epidural hematoma	0.19%—0.32%	Preoperative coagulation tests, the use of blunt-tipped puncture needles to reduce the risk of vascular injury, close monitoring, early recognition and management, and surgical removal of larger hematomas are essential.	Takawira et al., 2012; Garg and Wang, 2023; West et al., 2023; Koushik et al., 2024
Intraoperative nerve injury	Infrequently	Intraoperative refinement and gentle maneuvering to avoid injury are fundamental. Remove the device if damage occurs.	Eldabe et al., 2016; Kleiber et al., 2016; Koushik et al., 2024

This table presents the occurrence probabilities of common complications associated with spinal cord stimulation, along with suggestions for prevention or treatment.

In addition to these complications, the use of SCS in patients with implanted permanent pacemakers and cardioverter-defibrillators raises concerns. The electrical impulses generated by SCS may induce severe bradycardia or even sudden cardiac arrest due to electrical disturbances that can suppress pacemaker output or cause abnormal discharges from the implanted cardioverter-defibrillator (Egger et al., 2019). However, clinical practice has shown that the implantation of pacemakers or cardioverter-defibrillators is not a contraindication for SCS use. Safe implantation of SCS in such patients may still be possible with strict programmed management, bipolar stimulation modes, and individual testing of each patient (Iyer et al., 1998; Ekre et al., 2003; Enggaard et al., 2010). Additionally, real-time electrocardiographic monitoring during the initial implantation and a long-term follow-up mechanism to dynamically assess device interaction effects are recommended. However, there are some limitations in the imaging assessment of patients undergoing SCS. The magnetic field of MRI induces electrical currents, posing a risk of thermal damage to patients with SCS devices (Walsh et al., 2015), and may also cause dislocation and functional impairment of the SCS device (Vasquez et al., 2020). This makes MRI examination a relative contraindication for patients undergoing SCS. In recent years, advancements in technology and the development of new materials have led to the introduction of MRI-compatible SCS systems, which have demonstrated safety for specific sequence scans. Clinical cases have reported the successful application of MRI-compatible SCS devices (Moens et al., 2012; Provenzano et al., 2016). Strict guidelines must be followed in clinical practice (Vasquez et al., 2020): patients must remove external components associated with the SCS device before undergoing an MRI. A static magnetic field of either 1.5 T or 3 T should be utilized. The maximum whole-body mean SAR is 2.0 W/kg per pulse sequence for a 15-minute scan at 1.5 T/64 MHz, as well as for a 15-minute scan at 3 T/128 MHz. At 3 T/128 MHz, during 15 minutes of scanning per pulse sequence, the maximum whole-body average SAR was 0.3 W/kg for the torso, while the maximum SAR for the rest of the body remained at 2.0 W/kg, with adjustments made to lower SAR in order to prevent excessive temperature increases.

In conclusion, to maximize safety, the clinical application of SCS must adhere to a strict risk management strategy. Preoperatively, spinal anatomy should be evaluated through imaging to exclude severe deformities or space-occupying lesions. Intraoperative image-guided techniques should be employed to minimize puncture-related complications. A multidisciplinary follow-up system should be established to optimize stimulation parameters and regularly monitor device function. Patient education is also critical, providing clear information on battery replacement and limitations on daily activities. Overall, the complication rate of SCS is manageable under standardized procedures, and relative contraindications can be addressed under specific conditions. This is promising for future developments, as the risk-benefit balance remains significant in the management of chronic refractory pain.

## Limitations

The therapeutic potential of SCS in relieving chronic pain after SCI is supported by mechanistic studies (Lind et al., 2008; de Geus et al., 2023). Its mechanisms of action include, but are not limited to, the gating theory, neurotransmitter modulation, anti-inflammatory effects, and potential neurorestorative functions. Although notable progress has been made in understanding the mechanisms of action of SCS in chronic pain management following SCI, many pressing scientific questions remain in this field.

The main bottleneck facing current research is the lack of ideal animal models that accurately mimic the pathological features of chronic pain after SCI in humans. Limitations primarily include an over-reliance on reflexive behavior, difficulties in distinguishing real nociception from spinal hyperreflexia, and a lack of quantification of the affective dimension of pain (Kramer et al., 2017). Because animals cannot report pain subjectively, researchers have mainly relied on evoked reflex behaviors (e.g., claw retraction in response to mechanical or thermal stimuli) to assess pain. However, these metrics only reflect abnormal pain and nociceptive hypersensitivity similar to those experienced by humans, failing to capture the affective component of pain (Vierck and Yezierski, 2015). Additionally, SCI itself can interfere with assessment; hyperreflexia after SCI may trigger strong motor responses to stimuli below the plane of injury, which do not represent true nociceptive perception (Biurrun Manresa et al., 2014). This is particularly evident in animals with complete spinal cord transection, where sensory conduction pathways have been interrupted, yet they may still exhibit pain-like behaviors, such as avoidance or vocalizations, due to autonomic abnormalities or spinal reflexes. These behaviors can easily be misclassified as nociceptive hypersensitivity (Kramer et al., 2017). Current studies have used partial SCI models, but neuropathic pain in humans is commonly associated with complete injuries, resulting in an incomplete match between animal models and clinical situations. Alternative methods, such as the mouse grimace scale (Langford et al., 2010), spontaneous behavioral tests (Negus et al., 2015; Gould et al., 2016), and conditioned positional preference (Little et al., 2015), are capable of assessing the affective or persistent state of pain; however, they generally struggle to pinpoint the source of pain and are susceptible to interference from non-specific factors (Kramer et al., 2017). Therefore, future studies are needed to develop more accurate assessment strategies that incorporate multimodal indicators to more realistically model the complex mechanisms of human SCI pain.

From a clinical research perspective, the available evidence still has several limitations. Most studies have a retrospective design and limited sample sizes, which affects the reliability of the findings to some extent. Additionally, there is notable heterogeneity among studies, including differences in baseline patient characteristics (such as degree of injury and disease duration), inconsistencies in SCS intervention protocols (such as stimulation parameters and electrode locations), and variations in the criteria for evaluating efficacy. These factors have made it difficult to directly compare the results of different studies. Furthermore, pain assessment primarily relies on subjective patient reports, such as the Visual Analog Scale, which may introduce measurement bias. The generally short follow-up periods also constrain a comprehensive assessment of the long-term efficacy and safety of SCS.

Based on the limitations of current studies, future work should focus on the following aspects: optimizing animal models by improving construction techniques and developing more accurate assessment strategies; conducting large-sample, multi-center RCTs with standardized intervention protocols and objective assessment indicators; establishing more precise patient stratification criteria to identify target populations that are most likely to benefit from SCS treatment; developing novel biomarkers to enable objective quantification of pain levels; and extending follow-up periods to 3–5 years to systematically assess the long-term efficacy and safety of SCS. These research advances will provide a more reliable evidence-based rationale for the clinical application of SCS in the management of chronic pain after SCI.

## Conclusion

SCS has demonstrated significant clinical translational potential in managing chronic pain after SCI. SCS is effective in relieving neuropathic and spasm-related pain, particularly for intractable pain with limited effects from traditional medications. It can improve patients’ quality of life and reduce the use of paroxysmal medications. Its analgesic mechanism involves various neuromodulatory processes, including gate control theory, neurochemical mechanisms, anti-inflammatory effects, and neuroplasticity regulation. SCS regulates the balance of excitatory and inhibitory neural signals, reducing the transmission of harmful signals by stimulating the dorsal columns and dorsal horn neurons in the spinal cord. Additionally, SCS can promote the release of inhibitory neurotransmitters, such as GABA and 5-HT, enhancing the endogenous analgesic function of the spinal cord. It can also reduce the activation of microglia and astrocytes following SCI and decrease the expression of pro-inflammatory factors, such as IL-1β, IL-10, and TNF-α, to alleviate the neuroinflammatory state and reduce abnormal neural discharges. Previous studies have indicated that SCS may promote neuronal axonal repair and regeneration, reorganize and restore neural circuits, and regulate neuroplasticity after SCI by facilitating the release of substances such as BDNF and NGF, which in turn reduces abnormal signaling associated with pain (Goganau et al., 2018; Bacova et al., 2022). This provides a new reference for chronic pain treatment after SCI.

Despite the remarkable clinical effects of SCS in chronic pain management after SCI, many urgent issues remain to be addressed. Current studies primarily focus on neuropathic pain and spasm-related pain, while fewer investigations have been conducted on injurious pain and phantom limb pain. The long-term effectiveness and safety of SCS are not yet fully established, and large-scale, long-term follow-up data are still lacking. The optimal stimulation parameters corresponding to different pain types have yet to be determined, and there is considerable variability in therapeutic responses among different patients. Future research should involve high-quality RCTs to systematically evaluate the long-term effects and to explore the mechanisms of SCS in greater depth, optimizing stimulation modes and improving the precision and individualization of treatment. The application of AI and machine learning technology is expected to enhance patient screening and treatment plans through data analysis, thereby improving clinical efficacy. Furthermore, advancing the multidisciplinary collaboration model will aid in refining the precision of SCS surgery, optimizing postoperative rehabilitation protocols, and enhancing overall patient outcomes. Animal experiments should be integrated with clinical studies to further elucidate the mechanisms of SCS and provide a more robust theoretical foundation for chronic pain treatment after SCI. With more extensive research, SCS is expected to become a standard approach for managing chronic pain after SCI, offering patients more stable and long-lasting pain relief.

## Additional files:

***Additional Table 1:*** *Classification and characteristics of SCS.*

***Additional Table 2:*** *Clinical manifestations of different segments in complete spinal cord injury.*

***Additional Table 3:*** *Clinical studies of SCS for the treatment of pain after SCI.*

***Additional Table 4:*** *Recommendations for the prevention and management of complications associated with spinal cord stimulation.*
